# How prepared are we for cross-border outbreaks? An exploratory analysis of cross-border response networks for outbreaks of multidrug resistant microorganisms in the Netherlands and Germany

**DOI:** 10.1371/journal.pone.0219548

**Published:** 2019-07-10

**Authors:** Jacklien H. J. Maessen, Jörg Raab, Manon Haverkate, Martin Smollich, Henriëtte L. G. ter Waarbeek, Renske Eilers, Aura Timen

**Affiliations:** 1 Department of Organization Studies, Tilburg University, Tilburg, the Netherlands; 2 Centre for Infectious Disease Control, National Institute for Public Health and the Environment, Bilthoven, The Netherlands; 3 University Hospital of Schleswig-Holstein, Lübeck, Germany; 4 Public Health Service Zuid Limburg, Heerlen, the Netherlands; 5 Athena Institute for Research on Innovation and Communication in Health and Life Sciences, VU University Amsterdam, Amsterdam, the Netherlands; Kent State University, UNITED STATES

## Abstract

**Background:**

The emergence and spread of multidrug resistant microorganisms is a serious threat to transnational public health. Therefore, it is vital that cross-border outbreak response systems are constantly prepared for fast, rigorous, and efficient response. This research aims to improve transnational collaboration by identifying, visualizing, and exploring two cross-border response networks that are likely to unfold during outbreaks involving the Netherlands and Germany.

**Methods:**

Quantitative methods were used to explore response networks during a cross-border outbreak of carbapenem resistant Enterobacteriaceae in healthcare settings. Eighty-six Dutch and German health professionals reflected on a fictive but realistic outbreak scenario (response rate ≈ 70%). Data were collected regarding collaborative relationships between stakeholders during outbreak response, prior working relationships, and trust in the networks. Network analysis techniques were used to analyze the networks on the network level (density, centralization, clique structures, and similarity of tie constellations between two networks) and node level (brokerage measures and degree centrality).

**Results:**

Although stakeholders mainly collaborate with stakeholders belonging to the same country, transnational collaboration is present in a centralized manner. Integration of the network is reached, since several actors are beneficially positioned to coordinate transnational collaboration. However, levels of trust are moderately low and prior-existing cross-border working relationships are sparse.

**Conclusion:**

Given the explored network characteristics, we conclude that the system has a promising basis to achieve effective coordination. However, future research has to determine what kind of network governance form might be most effective and efficient in coordinating the necessary cross-border response activity. Furthermore, networks identified in this study are not only crucial in times of outbreak containment, but should also be fostered in times of non-crisis.

## Introduction

Modern societies face a renewed threat to transnational public health since an increasing number of bacteria have developed multidrug resistance to antibiotics. Prevalence rates of these multidrug resistant microorganisms (MDRO) are increasing [1,2], which contributes to an elevated outbreak risk. As a substantial number of patients cross national borders to receive medical treatment, and this number is expected to increase in the future [3,4], potential cross-border outbreaks of MDRO are a major hazard for international infection prevention and control.

Cross-border outbreaks have occurred regularly in various parts of the world, such as the 2009 flu pandemic [5], the 2013–2016 Ebola outbreak in West-Africa [6] and the 2011 *E*. *coli* outbreak in the German-Dutch border area [7]. Until now, according to the authors’ knowledge, no cross-border MDRO outbreak has been reported in real life, but dissemination of resistant microorganisms and the risks of outbreaks across borders have been described in previous publications [4,8]. Since treatment options for infections caused by MDRO are becoming increasingly limited, it is vital that outbreak response systems are constantly prepared for fast, rigorous, and efficient response in order to minimize the negative impact of potential outbreaks of MDRO and to prevent further transmission of the resistant microorganism [9,10].

Evaluations of previous (inter)national outbreaks have shown that these, like other complex problems, cannot be tackled by any individual organization on its own but instead need to be addressed jointly by multiple organizations through interorganizational networks [11–13]. Collaboration and effective coordination between affected health care institutions and other stakeholders in outbreak containment is crucial since it leads to increased capability to address the problem and allows a broader set of resources to be used [14–16].

In this study we apply a network perspective to response systems for outbreaks of MDRO involving the Netherlands and Germany. In this perspective, response systems are conceptualized as interorganizational networks which consist of a set of organizations or actors and the relationships between them, that pursue joint goals [17]. In this study, a response system is therefore conceptualized as a goal-directed inter-organizational network that emerges during outbreaks of MDRO in order for organizations to jointly reach rapid and thorough outbreak control. Being prepared for a coordinated response involving all affected institutions as well as other stakeholders in the outbreak response is essential to minimize the disease burden which may even include human deaths.

Conceptually, the cross-border response network can further be regarded as a web of nodes and ties, where nodes are the stakeholders that are involved in the outbreak response and ties are the collaborative relations that connect or separate them. Previous research in disaster management shows that social network analysis is a valuable technique in understanding, mapping, and applying characteristics of networks [18]. In social network analysis, emphasis is laid on the overarching structure of the relations between nodes since the configuration of the network has important implications for the effectiveness of the network [19,20]. With this sort of analysis, insights in potential coordination challenges and opportunities that networks might face can be gained and this will potentially contribute to the realization of better public health outcomes [18,21].

The present research aims to improve transnational collaboration in outbreak containment by identifying, visualizing, and exploring two cross-border response networks. These networks are likely and desired to emerge during potential cross-border outbreaks of MDRO involving the Netherlands and Germany. Outbreaks of MDRO have only recently become a threat to public health and an actual cross-border outbreak between the Netherlands and Germany has not yet occurred. Therefore, little is known about how to reach effective and efficient outbreak control in the preparation and response phases. This is especially the case with regard to whom will participate in the network, what the collaborative structures will look like, and how mutual actions consequently can best be coordinated.

## Materials and methods

### Questionnaire

An online questionnaire was set out to gather network data about the collaboration structures between stakeholders during a cross-border outbreak of MDRO involving the Netherlands and Germany. The questionnaire introduced a fictive, but realistic, cross-border outbreak scenario of MDRO. The respondents subsequently reflected on their professional roles and collaborative relationships with partners in the containment of the outbreak in the scenario. The questionnaire was set out in two cross-border regions, each consisting of a Dutch and a German neighboring municipal health service (MHS).

**Fig 1:**
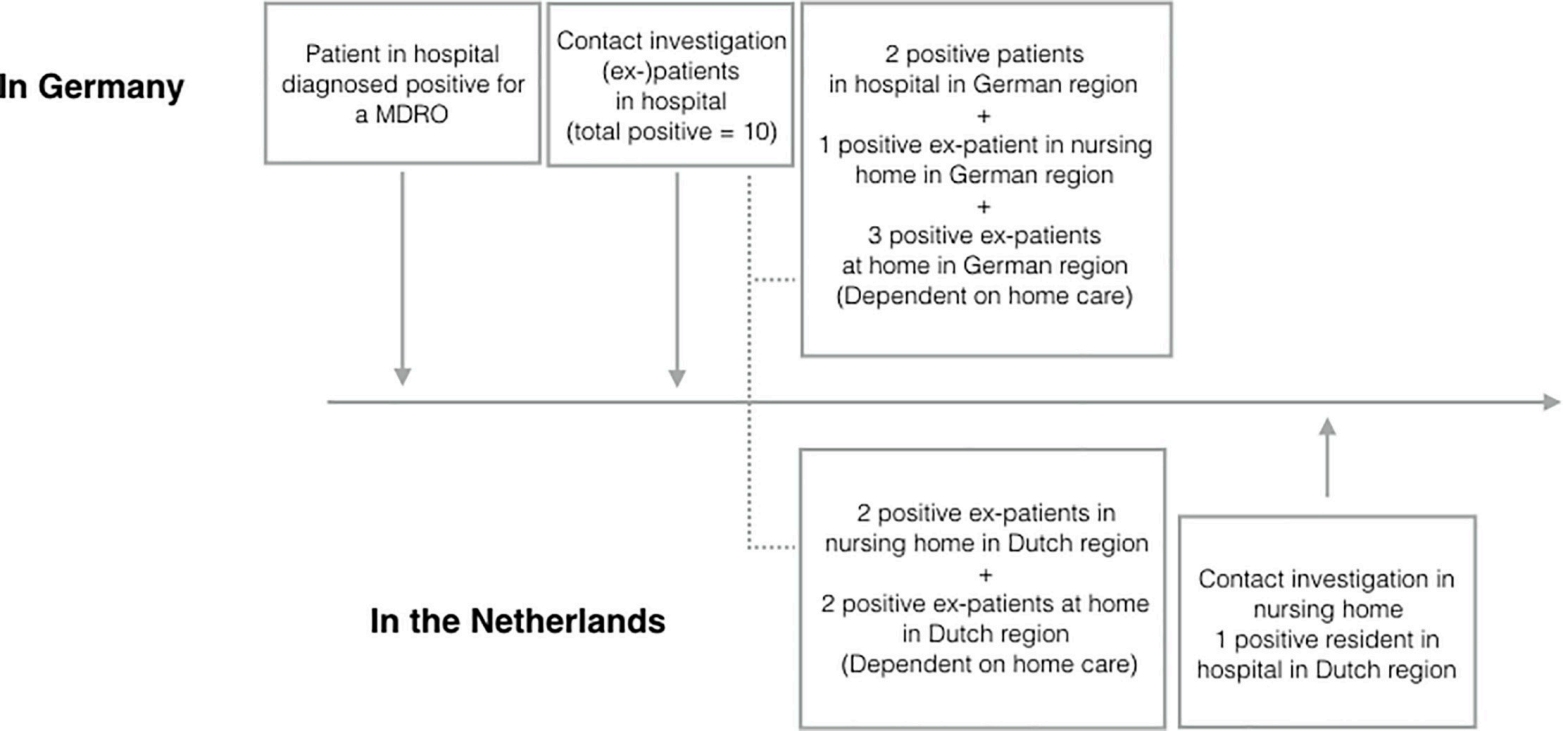
The fictive cross-border outbreak scenario.

In the outbreak scenario, carbapenem resistant Enterobacteriaceae (CRE) caused an infection in a patient in a German hospital and where (via patient referral) the CRE had spread to patients in several other institutions on both sides of the border. The scenario was based on a regional outbreak requiring collaboration between various healthcare organizations. This regional outbreak was adapted to fit the Dutch-German cross-border setting and was reviewed by experts. Fig 1 provides a schematic overview of the scenario.

The questionnaire was adopted from a previous study by the Dutch national institute for public health and the environment, which measured Dutch regional outbreak response networks [22]. The questionnaire was adjusted to a cross-border situation while keeping it as close to the original as possible. This was done to preserve the internal validity of the scenario since it was previously validated in a focus group with medical specialists. The questionnaire, which was originally in Dutch, was also translated to German. Before administering the questionnaire to the targeted sample, it was pretested by five infectious disease experts (three in the Netherlands and two in Germany) to make sure that any changes made to the questionnaire were correct (for example that the correct German medical terminology was used). The questionnaire was approved by the ethical review board of Tilburg University School of Social and Behavioral Sciences (EC-2017.43). The Dutch and German questionnaires are respectively available in S1 File and S2 File. A translated English version of the questionnaire can be found in S3 File.

**Fig 2:**
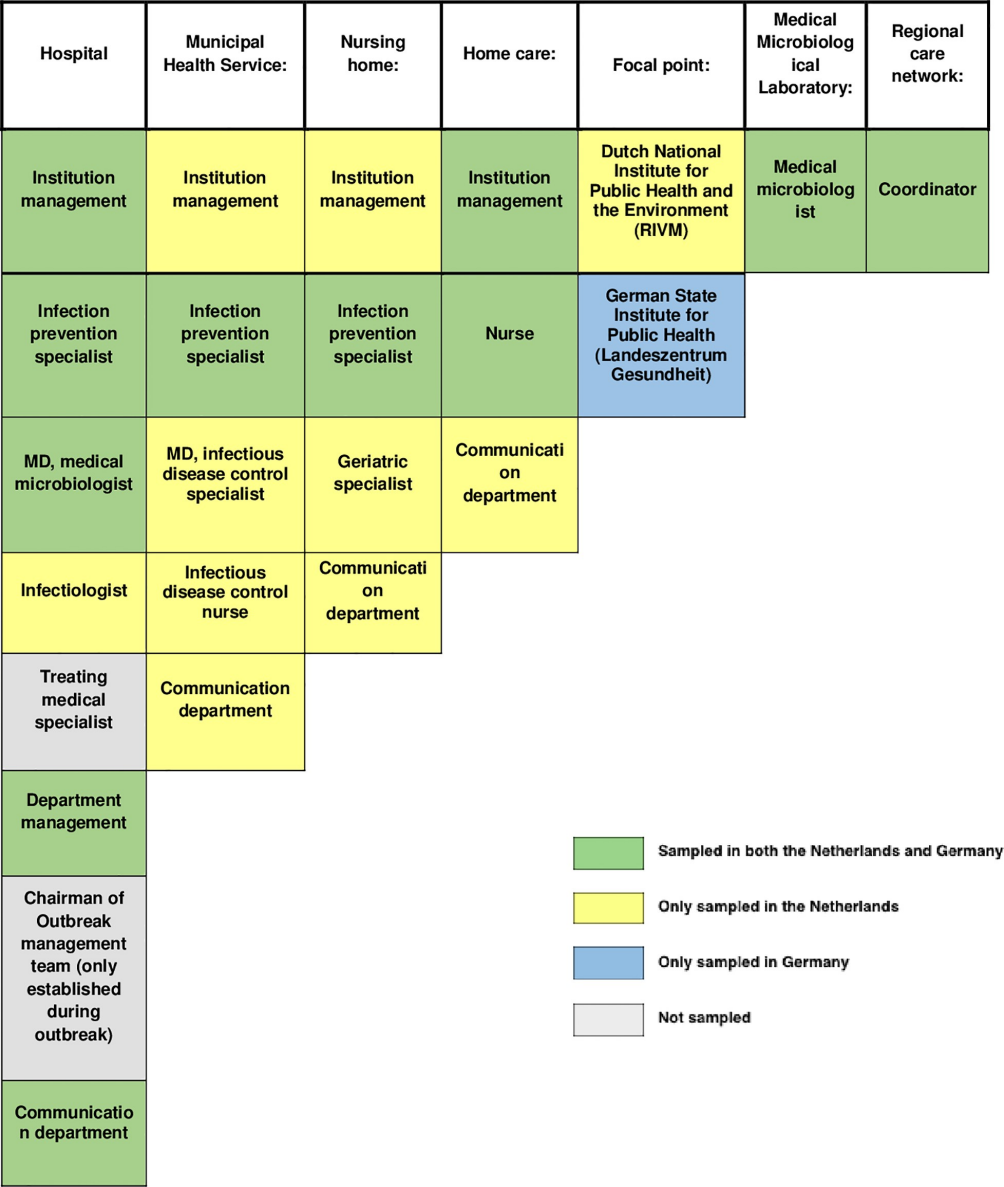
The set of stakeholders a priori identified by infectious disease experts.

Collaborative relations between stakeholders were measured using a *roster choice method* [23]. Multiple statements were presented, each along with a predefined list (roster) of potential stakeholders. Participants could select the stakeholders in the roster to which the statement applied. The statements asked the respondent to indicate 1) to whom they would give information during outbreak response, 2) from whom they would receive information, and 3) with whom they maintained prior working relationships. The roster of potential stakeholders was created through exploratory interviews with Dutch and German infectious disease experts. Since the health care systems in Germany and the Netherlands differ significantly, different stakeholders were identified per country. Fig 2 gives an overview of the complete set of stakeholders that were identified. For each statement the participants had the opportunity to mention additional stakeholders to which the statement applied, that were not in the roster, in an open answer field. Due to this, additional stakeholders potentially had a chance to be added to the network.

In addition, the questionnaire also measured general trust that the actors had in the response network via three items which were adopted from the before mentioned study by de Vries and colleagues [22]. Participants were asked to rate their agreement on a five-point scale to the following items: 1) I think that the other healthcare professionals involved have sufficient capacities to act satisfactory to this outbreak. 2) I think that the other healthcare professionals involved will prioritize collective outbreak response interests over personal- or institutional interests. 3) I think that the other healthcare professionals involved have the same ideas to mine concerning the right approach to this outbreak.

### Data collection

The data was gathered in two cross-border networks (CBA and CBB) both consisting of a Dutch and a German neighboring region. These four specific regions were chosen because data collection was most feasible there and because they are comparable in size and number of inhabitants. Although two specific cross-border networks were investigated in this research, the aim is to acquire knowledge which is generalizable to the general Dutch-German border area. This because the border area is relatively small (≈ 350 kilometers) and health systems do not differ substantially between regions on each side along this border.

The questionnaire was administered via e-mail to the health professionals that were a priori identified by Dutch and German infectious disease experts to be potential stakeholders in the outbreak scenario. All participants agreed with a written informed consent. In each region, the online questionnaire was sent to multiple professionals per stakeholder role. This way, the individual responses could be aggregated to the corresponding role in order to make the findings more representative, robust, and generalizable. E-mail addresses of the targeted health care professionals in the four regions were gathered in cooperation with the corresponding MHS.

### Data analysis

The relational data gathered via the roster choice statements were translated in adjacency matrixes. An adjacency matrix is a grid with the names of the actors displayed both the x-axis and y-axis. The cells in the matrix contain either a 1 (representing a relation between actors) or a 0 (representing the absence of a relation between actors) [23]. The adjacency matrixes are the basis for network visualization and analysis, which were done using the social network analysis software packages UCINET [19] and Visone [24].

Information sharing ties between network actors were visualized to represent the main collaborative structures during outbreak response. Focusing on the networks as a whole, network structure is conceptualized as the type and degree of integration that is present [25]. In this study we analyzed density, centralization and clique overlap as different types of network integration. In addition, we analyzed the similarity of the tie constellations between two networks with the Quadratic Assignment Procedure (QAP) correlations (all the measures will be further explained in the result section). At the level of individual nodes, centrality and brokerage measures were calculated. Density was calculated for the prior relationships networks and descriptive statistics were calculated for the trust items.

## Results

In total, 86 individual health professionals filled in the questionnaire. In cross-border region A, 25 out of the 33 targeted stakeholder roles were represented (76% response rate). In cross-border region B, 22 stakeholder roles were represented (65% response rate). A table indicating the exact number of respondents per stakeholder role is provides in S1 Table.

### Network visualization

**Fig 3:**
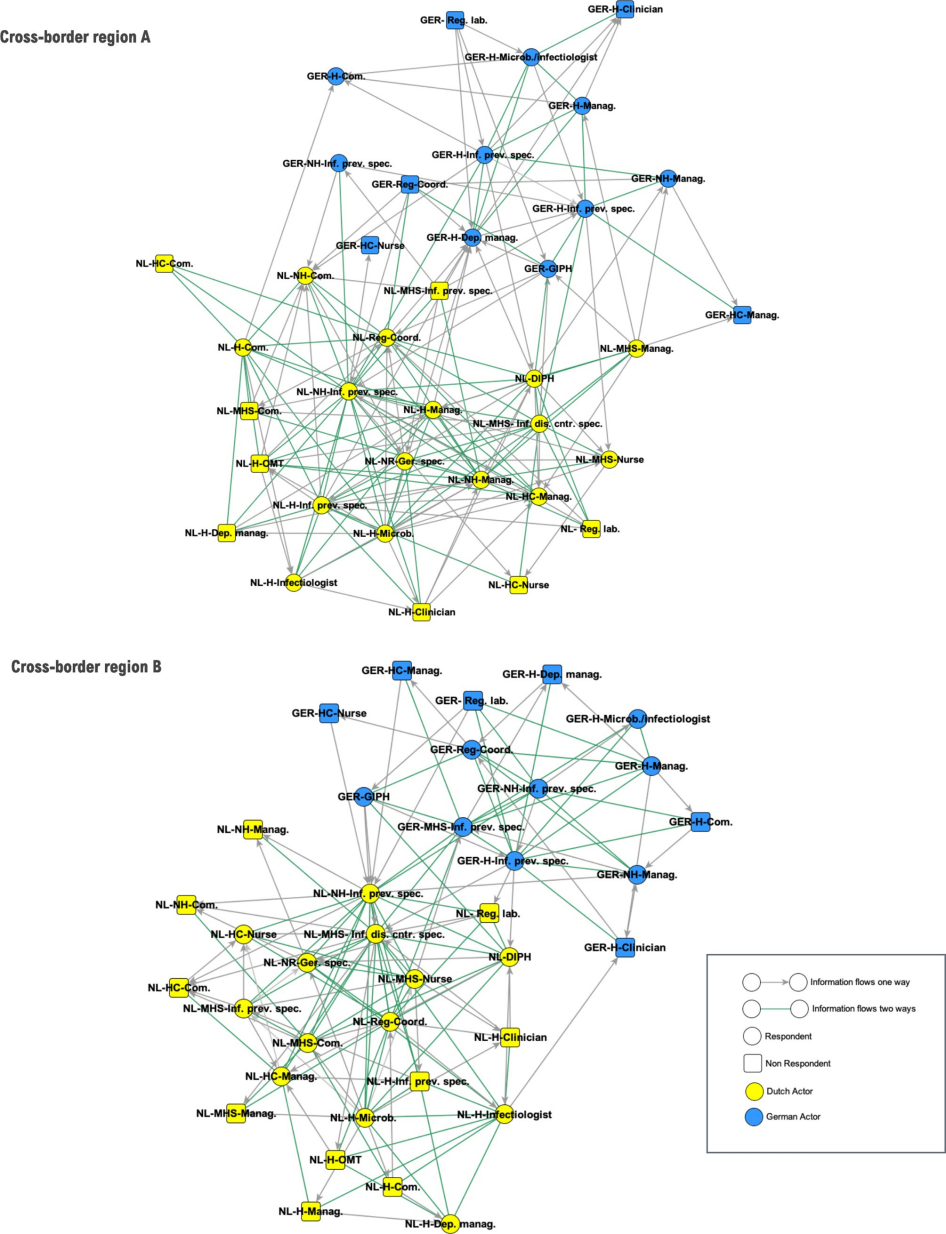
Information sharing network during outbreak response in cross-border region A and B.

The relational data acquired via the two information exchange roster choice items (to whom would you give information during outbreak response/ from whom would you receive information during outbreak response) were visualized in information sharing networks. These networks represent the main collaborative structures during a crisis in the cross-border response networks. The identified cross-border networks (CBA and CBB) can be found in Fig 3 (the labels are explained in S1 Table). In both cross-border regions, each stakeholder that was on the predefined list, was selected at least once in one of the two information sharing roster choice items. Hence, each stakeholder presented in Fig 2, was indicated by at least one respondent as information exchange partner in an effort to jointly reach effective outbreak control. No relevant additional stakeholders were mentioned by the participants in the open answer categories. Therefore, the survey results validated the predefined list of stakeholders, without adding additional stakeholders. This makes both cross-border response networks consist of the 37 predefined actors.

The position of each node in the visualizations of each network (see Fig 3) is determined by an algorithm called *spring embedder*. This algorithm positions nodes that are connected to each other closer together while it repels nodes which are less connected. When visually inspecting the networks it is evident that in both CBA and CBB, two subgroups are present which consist on the one hand of Dutch and on other hand of German actors. Despite this, the two subgroups are bridged via ‘cross-border ties’, which makes that the set of nodes form one cross-border response network as a whole.

### Network analysis

Three measures of network integration were used at the network level in order to explore to what extent the cross-border networks were integrated. Namely, *density*, *centralization*, *and clique structures*. These measures were selected because they have been shown to be important measures of network integration [26–28].

First, density indicates how many ties are present in a network as a proportion of the number of ties that can theoretically exist. It thus indicates the level of interconnectedness between actors [19]. The density of CBA was 0.197 which was slightly higher than that of CBB, which was 0.177 (see Table 1). This means that respectively 19.7% and 17.7% of the ties that could theoretically exist were present. Since 37 stakeholders from different institutions and countries share information with each other, we qualify this as moderate interconnectedness between the actors.

**Table 1:** Measurements of the information sharing (sub)networks and prior working relationships of the stakeholders.

	CBA	CBB	D subgroup CBA	G subgroup CBA	D subgroup CBB	G subgroup CBB	Transnat CBA	Transnat CBB
**Density information sharing ties**	0.197	0.177	0.364	0.242	0.304	0.335	0.053	0.033
**Degree centralization**	0.486	0.403	0.548	0.321	0.518	0.500	N/A	N/A
**Clique overlap**	6%	3%	17%	50%	13%	10%	21%	45%
**Density of prior working relations**	0.217	0.186	0.390	0.484	0.296	0.423	0.010	0.033

Since density is best interpreted in a comparative way, the density scores of the Dutch and German subgroups and the transnational ties within the cross-border networks were calculated (see Table 1). As can be seen in this table, density scores of the national subgroups within the cross-border regions were higher than the overall density of the cross-border networks. This means that national subgroups were more interconnected than the complete networks. Especially, the density of the cross-border ties was relatively low. There were only 34 ties that spanned national border in CBA (density of 0.053) and 21 in CBB (density of 0.033). Information sharing occurs mostly within national subgroups and least across national borders.

Secondly, Freeman’s degree centralization was calculated in order to indicate to what extent the network ties are centralized around one or a few actors [29]. This measure compares the centralization of a network to the centralization of a star graph. Hence, it indicates whether the number of ties that actors maintain is equally distributed among the network, or that certain (central) actors have more ties than others [30]. The following equation is used to measure degree centralization [29]:

$$C_{X}=\frac{\sum_{i=1}^{n}\left[C_{X}(p^{*})-C_{X}(p_{i})\right]}{max\sum_{i=1}^{n}\left[C_{X}(p^{*})-C_{X}(p_{i})\right]}$$

In this equation C_X_(p_i_) is a degree centrality (or the relative number of ties) of an actor. C_X_(p*) is the largest degree centrality in the network. Thus, the C_X_ index indicates the degree to which the largest degree centrality exceeds the degrees of other actors. This index can vary from 0 to 1. When the network is a star graph where one actor is dominating the network then C_X_ is 1. When C_X_ is 0 all degree centralities in the network are equal.

In their seminal article, Provan and Milward [27] argued that in large networks, centralization is beneficial for network effectiveness (i.e. the achievement of network level goals [17]) because it makes coordination more efficient compared to a decentralized structure. Degree centralization was chosen to measure the degree of integration of the networks rather than other centralization measures such as betweenness- and closeness centralization. This because degree centrality is a local measure and therefore less influenced by missing data due to lower response rates (<80%) as was the case in this study [31].

As can be seen in Table 1, the degree centralization of CBA was 0.486 and that of CBB was 0.403. Similar to the density scores, the centralization measures of both networks were relatively equal. Degree centralization of the national subgroups was also roughly comparable to these values, except for the somewhat lower centralization in the German subgroup in CBA. Regarding degree centralization, and looking at the sociogram in Fig 3 we can observe, that certain actors occupied more central roles than others and that the networks were organized around particular actors rather than including all actors equally.

Third, clique analysis was performed in order to reveal to what extent the networks were integrated via overlapping subsets or cliques. Cliques are defined here as groups of four or more actors that each are directly linked to each other [27]. Clique overlap represents a more decentralized form of integration compared to network centralization but does not require as many linkages as in case of integration through density [28]. The results showed that a large number of cliques consisting of a minimum of four actors could be found in the cross-border networks (respectively 48 and 44). Almost all actors were represented in at least one clique.

The overlap between cliques was calculated for each type of clique separately. From Table 1, it can be seen that in the cross-border networks as a whole, there was almost no overlap between the cliques. This implies that members belonging to a clique interacted amongst themselves, but to a lesser extent to members of other cliques. However, the overlap in cross-border cliques was fairly high (although higher in CBB than in CBA). This shows that a certain group of actors was mainly connecting the two national subgroups in the cross-border networks. When we specifically looked into these ‘border spanning’ cliques, it appeared that for CBA, the German state institute for public health (GIPH), the Dutch national institute for public health (DIPH), and the Dutch regional care network were these ‘integrating’ actors that appeared in more than half of the cross-border cliques. For CBB, these were the Dutch MHS infectious disease control specialist, the German MHS infection prevention specialist, the GIPH, the DIPH, and the Dutch nursing home infection prevention specialist.

### Comparing collaborative structures

Similarity and association between the tie constellations of the two cross-border networks could be compared using a technique called Quadratic Assignment Procedure (QAP) correlation [23]. QAP correlation is used rather than a more common Pearson’s r correlation because we want to correlate adjacency matrices that lay on the basis of the networks. In these matrices the basic statistical assumption of independence of observations is violated, since our observations (the ties in the network) are per definition not independent. QAP therefore works with a permutation test to deal with the non-independence of observations. This test calculates all the ways that links could have occurred in the networks and then counts the proportion of random assignments yielding a correlation as large as the one actually observed. The general logic therefore is that we want to compare the observed correlation against the distribution of correlations that one could obtain if the tie constellations between the actors of the two networks were in fact independent of each other [32]. The QAP correlation between the two networks was 0.395, which is statistically significant at the 5% level. This means that there is a moderate correspondence between the configuration of information sharing ties in response network A and B. The actors that exchange information during outbreak response in CBA are partly also the actors that exchange information in CBB.

### Identifying influential network actors

To identify influential network actors, degree centrality and brokerage calculations were done. These are properties of nodes rather than networks as they indicate a node’s relative position within a network. Since we are interested in identifying actors that are structurally suited to take leading roles, we want to identify those actors that are central in the information flow. For this reason, d*egree centrality* is calculated. Degree centrality counts the total number of ties that a node has (without regarding their direction) and divides this by the maximum number of ties it theoretically can have [29]. The higher the number of information sharing ties a node maintains, the more central it is in the information flow.

**Fig 4:**
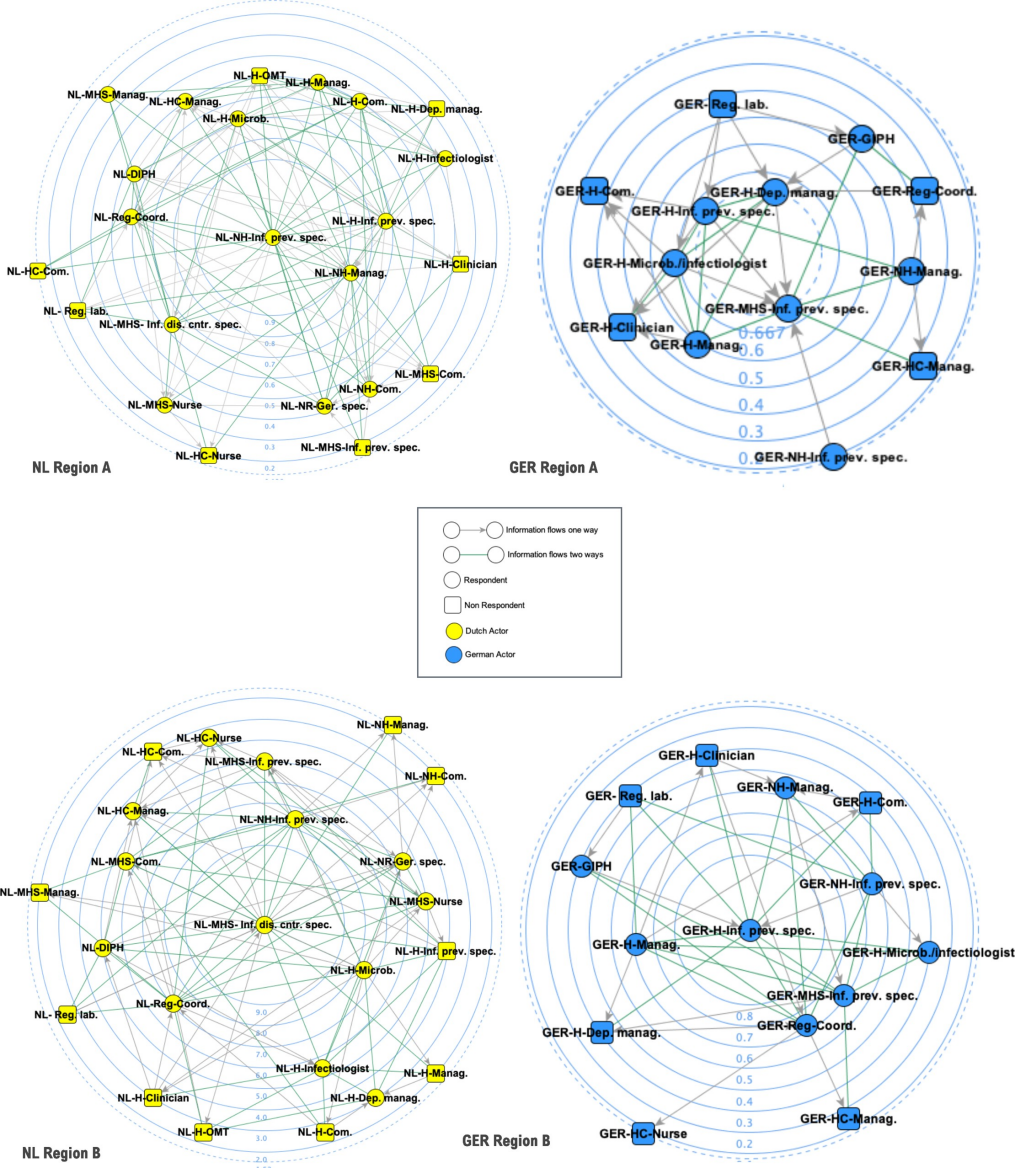
Sociogram of degree centrality per national cluster per cross-border information sharing network.

Fig 4 graphically illustrates the standardized degree centrality scores of each actor relative to the other actors in its region. The concentric circles in the background represent the exact degree centrality scores. Nodes with higher degree centrality are placed more in the center, while nodes with a lower degree centrality are positioned more peripherally (S2 Table contains the exact scores). From Fig 4, it can be seen that the MHS infectious disease control specialist, the hospital medical microbiologist and the nursing home infection prevention specialist and nursing home institution management are the actors with the highest degree centrality in the Dutch subgroups. The most central actors in the German subgroups were the MHS infection prevention specialist, the hospital infection prevention specialist, the regional care network and the hospital department management.

It is also important to identify actors that are influential in connecting different subparts of the networks. Therefore, *network brokers* were identified. Brokers are seen here as actors that link other actors who would otherwise be less or not connected. Since we are investigating cross-border networks, each actor in the network belongs to a distinct national group. Therefore, we were able to segment brokers into particular types as distinguished by Gould and Fernandez [33]. Via these brokerage measures implemented in UCINET, we particularly identified actors that broker between other actors who belong to different countries.

Hence, cross-border brokers were identified by using two types of brokers namely the *representative* and the *gatekeeper* brokers [33]. A representative broker receives information from its own national group and has the potential to transmit it to the other national group [34]. It is thus delegated to diffuse information across the border. A gatekeeper broker receives information from the other national group and can further diffuse it in its own national group [34]. For each node it was counted how many times it occupied a particular role. Note that it is also possible for a specific actor to occupy both brokerage roles. Identifying brokers that connect the national subgroups is important since they are potentially suited to coordinate the cross-border response.

Table 2 shows the number of times each actor occupied each brokerage role. As can be seen in Table 2, the following actors most often occupied broker roles: The Dutch nursing home infection prevention specialist, the Dutch MHS infectious disease control specialist, the German MHS infection prevention specialist, the German hospital infection prevention specialist, the DIPH, and the Dutch regional care network. The GIPH was also found to have a brokering role, but less than the other actors and only in CBA.

**Table 2:** Counts for the representative and gatekeeper brokerage roles.

	CBA Gatekeeper	CBA Representative	CBA Subtotal	CBB Gatekeeper	CBB Representative	CBB Subtotal	Trandtotal
NL nursing home—geriatric specialist	72	53	**125**	86	33	**119**	**244**
NL municipal health service—medical doctor, infectious disease control specialist	22	21	**43**	46	11	**57**	**100**
GER municipal health service—infection prevention department	7	15	**22**	27	16	**43**	**65**
NL focal point—dutch national insitution for public health	18	16	**34**	14	7	**21**	**55**
NL regional care network—coordinator	13	22	**35**	10	9	**19**	**54**
GER hospital—infection prevention specialist	7	15	**22**	0	30	**30**	**52**
GER hospital—department management	29	0	**29**	0	0	**0**	**29**
GER focal point—german state institution for public health	7	9	**16**	3	6	**9**	**25**
NL municipal health service—institution management	0	13	**13**	0	0	**0**	**13**
NL hospital—infectiologist	0	0	**0**	0	11	**11**	**11**
NL nursing home—communication department	11	0	**11**	0	0	**0**	**11**
NL hospital—communication department	0	10	**10**	0	0	**0**	**10**
NL hospital—infection prevention specialist	0	9	**9**	0	0	**0**	**9**
GER nursing home—institution management	7	0	**7**	2	0	**2**	**9**
GER nursing home—infection prevention specialist	1	0	**1**	5	3	**8**	**9**
NL hospital—institution management	0	8	**8**	0	0	**0**	**8**
NL municipal health service—infectious disease control nurse	3	0	**3**	0	5	**5**	**8**
GER hospital—institution management	6	0	**6**	0	0	**0**	**6**
NL hospital—medical doctor, medical microbiologist	0	5	**5**	0	0	**0**	**5**
NL municipal health service—infection prevention specialist	0	5	**5**	0	0	**0**	**5**
GER regional care network—coordinator	1	4	**5**	0	0	**0**	**5**
GER hospital—treating medical specialist	0	0	**0**	3	0	**3**	**3**
GER homecare—institution management	1	0	**1**	0	1	**1**	**2**
GER medical microbiological laboratory—medical microbiologist	0	0	**0**	0	2	**2**	**2**
GER homecare—nurse	0	0	**0**	0	1	**1**	**1**
NL hospital—chairman of outbreak management team	0	0	**0**	0	0	**0**	**0**
NL hospital—treating medical specialist	0	0	**0**	0	0	**0**	**0**
NL hospital—department management	0	0	**0**	0	0	**0**	**0**
NL municipal health service—communication department	0	0	**0**	0	0	**0**	**0**
NL homecare—institution management	0	0	**0**	0	0	**0**	**0**
NL homecare—nurse	0	0	**0**	0	0	**0**	**0**
NL homecare—communication department	0	0	**0**	0	0	**0**	**0**
NL nursing home—institution management	0	0	**0**	0	0	**0**	**0**
NL nursing home—infection prevention specialist	0	0	**0**	0	0	**0**	**0**
NL medical microbiological laboratory—medical microbiologist	0	0	**0**	0	0	**0**	**0**
GER hospital—medical microbiologist/infectiologist	0	0	**0**	0	0	**0**	**0**
GER hospital—communication department	0	0	**0**	0	0	**0**	**0**

### Trust and prior working relationships

Trust between network actors is measured because many (network) scholars view it as the glue that holds networks together and that facilitates cooperation [12]. Trust is an attribute of a relationship that can be defined as “the willingness to accept vulnerability based on positive expectations about another’s intentions or behaviors” ([35] p. 92). Disaster management literature acknowledges the importance of trust and stresses that under uncertain environmental conditions and pressing urgency (under which the response network operates), trust between actors is needed in order for them to share information and resources with each other and to properly allocate responsibilities [13]. For each trust item, roughly 30% of the respondents indicated that they neither agreed nor disagreed with the statement. This indicates that there was quite some inconclusiveness about trust in the general network. The item stating that other actors would prioritize collective response above institutional interests yielded a divided response where one third of the respondents answered positively and the other third answered negatively. The items about other actors having sufficient capacities and similar ideas were predominantly answered positively (57% and 51%).

Since familiarity and trust take time to develop, they are difficult to build in times of an unexpected outbreak response. Therefore, the presence or absence of prior working relationships between network actors is measured. In this study, we examined to what extent prior working relationships are present and how they are distributed throughout the cross-border response networks [17].

The density of prior relationships was calculated to explore how they were distributed among the prior-working relationship networks (Table 1). For both networks, the national subgroups are more densely connected than the overall networks which confirms that more prior relationships are present between actors belonging to the same country than to different countries. When focusing specifically on prior relations that spanned national borders, it becomes clear that these are sparse. In CBA, only four actors are involved in border spanning ties. Only the DIPH, the GIPH, and the two MHS are involved in cross-border working relations. In CBB, we observed more border spanning relations, but they remain sparser than in the collaborative networks during outbreak response.

## Discussion

This research was set out to map and potentially improve transnational collaboration in outbreak containment by empirically identifying, visualizing, and exploring two cross-border response networks that are likely and desired to emerge during potential cross-border outbreaks of MDRO involving the Netherlands and Germany.

Based on a fictive but realistic outbreak scenario, our comparative case research revealed two relatively similar regional cross-border MDRO response networks in which 37 stakeholders were involved in joint outbreak response. We found that the structure of the response networks was highly clustered into national subgroups. Collaboration was much more frequent within countries than between them. However, despite this national clustering, we can speak of relatively integrated cross-border response networks since the national subgroups were connected or brokered by a small group of stakeholders. Hence, integration between Dutch and German stakeholders in this scenario was reached in a centralized manner rather than through numerous transnational collaborative ties.

The clustered network structure allows us to distinguish between national and cross-border authority roles. Within countries, we saw a centralized network structure with potential leading roles for the MHS and the regional hospital, as they were most central in the information flow during outbreak response. Across national borders, we could identify several stakeholders who were beneficially positioned to coordinate transnational collaboration, namely: both the German and Dutch MHS, both national/state focal points (the DIPH and GIPH), both hospital infection prevention specialists, the Dutch regional care network, and the Dutch nursing home infection prevention specialist. This group of actors is multidisciplinary, as actors from health care and public health are involved.

Our findings clearly show that there is a strong potential for network-level coordination. However, levels of general trust are moderately low and cross-border prior working relationships are sparse. It appears that although stakeholders within countries know each other, most of the stakeholders from different countries that ought to collaborate during a potential cross-border outbreak do not know each other prior to outbreak response. Previous publications have shown the importance of mutual trust and understanding when collaboration crosses national borders, with respect to mitigating the threat of outbreaks [36,37].

### Implications

A cross-border outbreak of resistant microorganisms would pose a coordination challenge for the involved health organizations on both sides of the border. The network analysis demonstrates that there is a good basis to achieve coordination of response activities across the border. However, it is not possible from the data at this point to determine how effective and efficient the coordination actually would be. In addition, we must take into account that a potential cross-border outbreak of MDRO is a low frequency high impact scenario and therefore the governance of response networks is inherently dynamic [38]. Attention should not solely be paid to governance in times of outbreak response (where the main task is containment), but the network should also be fostered in times of non-crisis, where the main task is anticipation of potential outbreaks. How collaboration in times of non-crisis should be arranged is out of the scope of the current research. However, for policymakers, it is important to take this into account when designing outbreak management guidelines or protocols. The results indicate that the greatest improvement can be gained in cross-border trust and prior relationships. The importance of prior relationships between stakeholders that are involved in emergency response is broadly shown by previous disaster management research [13,38]. Preparedness can be improved by organizing meetings where actors who need to collaborate during outbreak response can meet and exchange ideas and working methods. Training and simulation exercises can also improve trust in the general network and should be held in a common language such as English, as language barriers may be at the root of sub-optimal prior working relationships.

### Strengths and limitations

Previous research argues that as large-scale public health emergencies are rare events, innovative methods are needed to assess and improve preparedness [39]. In a rather novel and multidisciplinary manner, the current study used social network analysis to analyze the structure of interorganizational collaboration during infectious disease outbreak response. Social network analysis is applied only once before in outbreak management, and the use of an ex ante scenario is new. Since a comparative case study design was applied, and the same stakeholders appeared to be involved in two regional cross-border networks, we can be confident that we identified all relevant stakeholders applicable in our outbreak scenario. Another strong suit of the research is that, contrary to other disaster management studies [13,40], it investigates coordination structures prior to an actual crisis based on a fictive scenario. In doing so, preparedness of response systems can be improved through ‘lessons learned’ prior to an actual outbreak occurring. The research identified two separate networks, which were despite completely independent data collection very similar in structure. This indicates that especially given the limited length of the border and very similar health care systems in each country along the border knowledge acquired through this study might be generalizable to the general Dutch-German border area.

Despite these strengths, the results of this research need to be interpreted in light of its limitations. The most apparent limitation is the moderate response rate of the survey. While response rates of 76% and 65% are commonly quite acceptable in organizational and public health research, network analysis is more sensitive to missing data. Therefore, a response rate of at least 75% is commonly considered sufficient to accurately visualize social network data [41]. In this research, non-response is partly balanced out by reciprocal nominations, which means that actors that did not fill in the questionnaire are still included in the network since other actors indicated to collaborate with them [42]. Also, we measured degree centrality which is a local network measure and therefore is less susceptible to non response than global measures such as betweenness- or closeness centrality [31,43].

As in every survey-based study, we cannot eliminate that survey bias may have played a role in our research. The health professionals that responded to the survey, could be more likely to communicate with others during outbreak response. Additionally, we could not get a response from the clinician and the hospital outbreak management team since it is not clear who they will be prior to an actual outbreak. Another limitation of the research is that, as argued in the disaster management literature, the configuration of a response network is highly contingent upon the specifics of the crisis situation at hand [44]. Since the current study was exploratory, we chose to investigate a broad potential outbreak where multiple institutions were affected. In addition, the study does not provide an assessment, how effective the response would be.

### Future research

Future research should combine social network analysis techniques with qualitative research methods to gain deeper insight in the environmental conditions in which response networks operate and to increase external validity for other cross-border contexts. Also, simulation studies would yield valuable insights in the coordination needs of response networks. As is true for assessing any complex phenomenon, using a mixed method approach is likely to lead to more robust findings [12]. Future research should also combine the network analysis with the question, which network governance forms are most suitable and assess their effectiveness and efficiency in the response. The results of the network analysis make two forms likely options, since the involved organizations are now the connectors between the two country clusters: first, a dual lead organization with the two regional health services from the Netherlands and Germany jointly coordinating the response. Second, one could however also think of a broader core group of organizations from both countries forming a steering group taking on the necessary coordination.

In addition, future research should pay attention to network dynamics. It should not only differentiate between anticipation and containment phases but should also be sensitive for network dynamics and evolution within periods of outbreak containment [18]. Longitudinal research is needed to assess how the cross-border response networks will evolve, especially after a potential outbreak occurs.

## Conclusions

This research responded to diverse calls to strengthen transnational collaboration in managing antibiotic resistance. In doing so, a network perspective was applied to cross-border response systems for outbreak of MDRO. The results of this analysis show that relatively integrated cross-border response networks can be expected to emerge in such a scenario. Within the networks we found a clear potential for network-level coordination. Although stakeholders in the outbreak containment mostly collaborate with other health care professionals belonging to their own country, transnational collaboration was present in a centralized manner such that vital information could potentially efficiently be diffused on both sides of the border. This study improved the understanding of cross-border response networks emerging during outbreaks of MDRO and their governance, which contributes to increased preparedness for a contemporary threat to transnational public health.

## Supporting information

S1 File Dutch questionnaire. (PDF)

S2 File German questionnaire. (PDF)

S3 File English translated version of the questionnaire. (PDF)

S1 Table The exact number of health professionals that responded to the survey per region. (XLSX)

S2 Table Standardized degree centrality scores of all actors. (XLSX)
