# Supplementary material for: How prepared are we for cross-border outbreaks? An exploratory analysis of cross-border response networks for outbreaks of multidrug resistant microorganisms in the Netherlands and Germany
Source: PLoS One. 2019 Jul 10;14(7):e0219548. doi: 10.1371/journal.pone.0219548 (PMC6619808; doi:10.1371/journal.pone.0219548)
Supplement: S1 File — (PDF) [file pone.0219548.s001.pdf]

Rollen en verwachtingen bij de bestrijding van cross-border BRMO uitbraken

Geachte meneer/mevrouw,

Hartelijk dank voor uw deelname aan het onderzoek 'Rollen en verwachtingen van stakeholders bij de bestrijding van grensoverschrijdende uitbraken van bijzonder resistente micro-organismen'. Dit is een onderzoek van de Landelijke coördinatie Infectieziektebestrijding (LCI) van het RIVM in samenwerking met Tilburg University.

Met deze vragenlijst willen wij meer inzicht krijgen in de verschillende rollen en verwachtingen van betrokken zorginstellingen en zorgprofessionals, ten tijde van een grens- en instellingsoverschrijdende uitbraak van bijzonder resistente micro-organismen (BRMO). Het doel van dit onderzoek is het in kaart brengen van een respons netwerk dat in werking treedt in het geval van een grensoverschrijdende uitbraak van BRMO in Nederland en Duitsland.

Uw deelname is belangrijk voor ons. De resultaten zullen bijdragen aan de ontwikkeling van richtlijnen voor BRMO uitbraakbestrijding. Het invullen van de vragenlijst duurt ongeveer 15 minuten. Het is mogelijk om uw antwoorden tussentijds op te slaan en het invullen van de vragenlijst op een later tijdstip te hervatten. Zoals u in het toestemmingsverklaringformulier op de volgende pagina kunt lezen, zullen uw antwoorden vertrouwelijk behandeld worden en is uw deelname vrijwillig.

Voor vragen over het onderzoek kunt u contact opnemen met Jacklien Maessen door te e-mailen naar jacklien.maessen@rivm.nl

Toestemmingsformulier

Via deze link kunt u de toestemmingsverklaring bekijken.

Bij deze geef ik aan dat ik de toestemmingsverklaring heb gelezen en begrepen. Ik begrijp het doel van het onderzoek en begrijp wat er van me gevraagd wordt. Ik begrijp dat ik mijn deelname aan dit onderzoek op ieder moment kan stoppen en dat ik kan besluiten om vragen niet te beantwoorden. Ik begrijp dat deelname vertrouwelijk is en dat er geen conclusies worden getrokken op basis van mijn individuele bijdrage. Ik begrijp dat de data van dit onderzoek volgens protocol tien jaar lang bewaard blijven in een beveiligd dossier. Ik geef hierbij aan dat ik de mogelijkheid heb gehad om vragen te stellen en dat ik vrijwillig deelneem aan dit onderzoek.

Bij deze ga ik akkoord met de toestemmingsverklaring:
❑ Ik ga akkoord met de toestemmingsverklaring (1) ____________________

Algemene gegevens

Er volgen nu 2 algemene vragen over u en de zorginstelling waarin u werkzaam bent.

In welke regio bent u werkzaam?
❍ GGD-regio X (1)
❍ GGD-regio Zuid-Limburg (2)

Waar bent u werkzaam en wat is uw functie?
Bent u in meerdere zorginstellingen werkzaam? Selecteer dan de zorginstelling waar via u bent aangeschreven voor deelname aan dit onderzoek.

- ❍ Ziekenhuis - Raad van Bestuur/Directie (1)
- ❍ Ziekenhuis - Deskundige Infectiepreventie (2)
- ❍ Ziekenhuis - Arts-Microbioloog (3)
- ❍ Ziekenhuis - Infectioloog (4)
- ❍ Ziekenhuis - Afdelingsmanager (5)
- ❍ Ziekenhuis - Communicatie Adviseur (7)
- ❍ GGD - Directeur Publieke Gezondheid (8)
- ❍ GGD - Deskundige Infectiepreventie (9)
- ❍ GGD - Arts Infectieziektebestrijding (10)
- ❍ GGD/Gesundheitsamt - Verpleegkundige (11)
- ❍ GGD - Communicatie Adviseur (12)
- ❍ Thuiszorg - Raad van Bestuur/Directie (13)
- ❍ Thuiszorg - Verpleegkundige/Verzorgende (14)
- ❍ Thuiszorg - Communicatie Adviseur (15)
- ❍ Verpleeghuis - Raad van Bestuur/Directie (16)
- ❍ Verpleeghuis - Deskundige Infectie Preventie (17)
- ❍ Verpleeghuis - Specialist Ouderengeneeskunde (18)
- ❍ Verpleeghuis - Communicatie Adviseur (19)
- ❍ Regionaal Laboratorium Medische Microbiologie - Arts-Microbioloog (20)
- ❍ Regionetwerk - Coördinator/Kwartiermaker (21)
- ❍ RIVM - LCI (22)
- ❍ Anders, namelijk: (23) ____________________

Het grens- en instellingsoverschrijdende BRMO uitbraak scenario

Hieronder vindt u een fictief scenario waarin een grensoverschrijdende uitbraak van bijzonder resistente micro-organismen in zowel Nederland and Duitsland wordt beschreven. Op basis van dit scenario zullen wij u een aantal vragen stellen.

Wij verzoeken u dit scenario zorgvuldig te lezen.

Sinds enkele dagen ligt mevrouw Schmidt (84 jaar en woonachtig in het Duitse Kalkar) in een ziekenhuis in de regio X op de afdeling 'kortdurige opnames' in verband met aanhoudende hoge koorts en lage bloeddruk. De behandelend arts constateert dat er sprake is van urosepsis.

Het antibioticum dat wordt toegediend (derde generatie cefalosporine) slaat niet aan. Het microbiologisch laboratorium toont na twee dagen kweek een infectie met een NDM-1 producerende Klebsiella pneumoniae in de urine van mevrouw Schmidt aan. Dit type Klebsiella pneumoniae is resistent voor co-trimoxazol en fluorchinolonen, maar gevoelig voor aminoglycosiden, tigecycline en colistine.

Er wordt een nieuwe anamnese uitgevoerd. Daaruit blijkt dat mevrouw Schmidt kortgeleden met gelijke klachten opgenomen is geweest in een ziekenhuis in Griekenland, maar dit niet heeft verteld bij

opname. Zij is na verbetering van de klachten teruggevlogen naar Duitsland. Na vier dagen thuis te zijn geweest werd zij echter opgenomen in het ziekenhuis.

Conform protocol wordt mevrouw Schmidt vanaf nu in isolatie behandeld. Bij haar huidige kamergenoten worden kweken afgenomen om te zien of zij drager zijn van deze NDM-1 producerende Klebsiella pneumoniae (ring-/contactonderzoek).

De uitkomst van dit eerste ringonderzoek is aanleiding om een groter ringonderzoek uit te voeren op de ziekenhuisafdeling. Op basis van de uitkomst van dit vervolgonderzoek op de afdeling, wordt er besloten alle (ex)-patiënten van de afdeling van mevrouw Schmidt sinds haar opname te screenen.

In totaal worden er 38 (ex-)patiënten gescreend op dragerschap van deze NDM-1 producerende Klebsiella pneumoniae. Tien van de gescreende (ex-)patiënten worden positief bevonden. Twee van deze patiënten bevinden zich nog in het ziekenhuis in regio X. Drie van de ex-patiënten met dragerschap zijn thuis in de regio X. Deze mensen bezoeken wegens hun langer bestaande gezondheidsklachten regelmatig het ziekenhuis en zijn daarnaast alle drie afhankelijk van thuiszorg bij hun dagelijkse verzorging. Eén positief bevonden ex-patiënt bevindt zich in een verpleeghuis in de regio X.

Twee van de positief bevonden ex-patiënten waren overgeplaatst naar een verpleeghuis in de nabijgelegen Nederlandse regio X. Uit vervolg ringonderzoek van het verpleeghuis blijkt dat één andere bewoner positief bevonden is en deze is ondertussen opgenomen in een ziekenhuis in X in verband met andere gezondheidsklachten. Daarnaast is één ex-patiënt met dragerschap thuis in de regio X. Ook deze persoon heeft onderliggend lijden en komt frequent in het ziekenhuis. Deze ex-patiënt is eveneens afhankelijk van thuiszorg.

Vanochtend kopt de regionale Nederlandse krant met "Duits ziekenhuis veroorzaakt uitbraak superbacterie in Nederland". De Duitse krant kopt "Uitbraak van superbacterie in regio X". Beide artikelen beschrijven het persoonlijk relaas van een van de patiënten en weiden uit over de groeiende risico's van resistente micro-organismen.

Later in deze vragenlijst, kunt u het scenario inclusief het schematische overzicht nog altijd herlezen door op deze link te klikken.

Introductie van vragen bij het fictieve grensoverschrijdende BRMO-scenario

Wij zullen u in drie delen vragen stellen over het grensoverschrijdende BRMO uitbraak scenario:
Deel 1 gaat over bestrijdings-activiteiten.
Deel 2 gaat over samenwerking tussen zorgprofessionals.
Deel 3 bestaat uit een aantal afsluitende stellingen over de uitbraakbestrijding.

Wij vragen u om iedere vraag te beantwoorden vanuit uw eigen functie in uw organisatie. Verder is het belangrijk om er bij het beantwoorden van de vragen vanuit te gaan dat de zorginstellingen of zorgprofessionals genoemd in het scenario betrekking hebben op u. U bent, binnen uw team van collega's, het aanspreekpunt bij deze uitbraak. Bijvoorbeeld, u bent arts in een Nederlands verpleeghuis en er wordt gesproken van een Nederlands verpleeghuis in het scenario; U kunt dan de aanname maken dat het uw verpleeghuis is dat betrokken is in dit scenario en dat u de verantwoordelijke arts bent.

U kunt nu beginnen met het eerste deel van de vragen op de volgende pagina.

Deel 1. Grens- en instellingsoverschrijdende uitbraak-activiteiten

Hieronder vindt u 15 bestrijdings-activiteiten die plaats kunnen vinden bij een grensoverschrijdende BRMO uitbraak. Kunt u voor ieder van deze activiteiten aangeven hoe waarschijnlijk het is dat u erbij betrokken bent in dit scenario?   Let op: Wanneer er gesproken wordt van 'de uitbraak' wordt hiermee de gehele grens-en instellingsoverschrijdende uitbraak, zoals beschreven in het scenario, mee bedoeld.

U kunt het scenario teruglezen door op deze link te klikken.

1a Het plaatsnemen in een outbreak managementteam/beleidsteam om beslissingen te nemen over de bestrijding van de uitbraak.

- ❍ Ik ben zeker betrokken (1)
- ❍ Ik ben waarschijnlijk betrokken (2)
- ❍ Ik weet het niet (3)
- ❍ Ik ben waarschijnlijk niet betrokken (4)
- ❍ Ik ben zeker niet betrokken (5)

1b Het screenen van ex-kamergenoten van mevrouw Schmidt die zich buiten het ziekenhuis bevinden (in een verpleeghuis of thuis).

- ❍ Ik ben zeker betrokken (1)
- ❍ Ik ben waarschijnlijk betrokken (2)
- ❍ Ik weet het niet (3)
- ❍ Ik ben waarschijnlijk niet betrokken (4)
- ❍ Ik ben zeker niet betrokken (5)

1c Het opschalen van de infectiepreventiemaatregelen (implementeren van aanvullende maatregelen) in het verpleeghuis.
- ❍ Ik ben zeker betrokken (1)
- ❍ Ik ben waarschijnlijk betrokken (2)
- ❍ Ik weet het niet (3)
- ❍ Ik ben waarschijnlijk niet betrokken (4)
- ❍ Ik ben zeker niet betrokken (5)

1d Het implementeren van infectiepreventiemaatregelen in de thuissituatie van de BRMO positief bevonden mensen (beschermende maatregelen bij lichamelijke verzorging).
- ❍ Ik ben zeker betrokken (1)
- ❍ Ik ben waarschijnlijk betrokken (2)
- ❍ Ik weet het niet (3)
- ❍ Ik ben waarschijnlijk niet betrokken (4)
- ❍ Ik ben zeker niet betrokken (5)

1e Voorlichting geven aan de BRMO positief bevonden mensen in de thuissituatie.
- ❍ Ik ben zeker betrokken (1)
- ❍ Ik ben waarschijnlijk betrokken (2)
- ❍ Ik weet het niet (3)
- ❍ Ik ben waarschijnlijk niet betrokken (4)
- ❍ Ik ben zeker niet betrokken (5)

1f Vragen beantwoorden van het algemeen publiek (niet te patiënten betrokken bij ringonderzoek) over de uitbraak.
- ❍ Ik ben zeker betrokken (1)
- ❍ Ik ben waarschijnlijk betrokken (2)
- ❍ Ik weet het niet (3)
- ❍ Ik ben waarschijnlijk niet betrokken (4)
- ❍ Ik ben zeker niet betrokken (5)

1g Patiëntgegevens uitwisselen tussen zorgprofessionals en zorginstellingen ten behoeve van de uitbraakbestrijding.
- ❍ Ik ben zeker betrokken (1)
- ❍ Ik ben waarschijnlijk betrokken (2)
- ❍ Ik weet het niet (3)
- ❍ Ik ben waarschijnlijk niet betrokken (4)
- ❍ Ik ben zeker niet betrokken (5)

1h Communiceren met de media over de uitbraak
- ❍ Ik ben zeker betrokken (1)
- ❍ Ik ben waarschijnlijk betrokken (2)
- ❍ Ik weet het niet (3)
- ❍ Ik ben waarschijnlijk niet betrokken (4)
- ❍ Ik ben zeker niet betrokken (5)

1i Het opzetten van/bijdragen aan een grens- en instellingsoverschrijdend casusregister van de uitbraak.
- Ik ben zeker betrokken (1)
- Ik ben waarschijnlijk betrokken (2)
- Ik weet het niet (3)
- Ik ben waarschijnlijk niet betrokken (4)
- Ik ben zeker niet betrokken (5)

1j Het evalueren van uitgevoerde acties en samenwerking bij de grens- en instellingsoverschrijdende uitbraakbestrijding.
- Ik ben zeker betrokken (1)
- Ik ben waarschijnlijk betrokken (2)
- Ik weet het niet (3)
- Ik ben waarschijnlijk niet betrokken (4)
- Ik ben zeker niet betrokken (5)

1k Het informeren van gemeentelijke bestuurders over de uitbraak.
- Ik ben zeker betrokken (1)
- Ik ben waarschijnlijk betrokken (2)
- Ik weet het niet (3)
- Ik ben waarschijnlijk niet betrokken (4)
- Ik ben zeker niet betrokken (5)

1l Melden van de grens- en instellingsoverschrijdende uitbraak aan de nationale autoriteiten (RIVM)
- Ik ben zeker betrokken (1)
- Ik ben waarschijnlijk betrokken (2)
- Ik weet het niet (3)
- Ik ben waarschijnlijk niet betrokken (4)
- Ik ben zeker niet betrokken (5)

1m Contact opnemen met collega's in Duitsland voor uitwisseling van informatie.
- Ik ben zeker betrokken (1)
- Ik ben waarschijnlijk betrokken (2)
- Ik weet het niet (3)
- Ik ben waarschijnlijk niet betrokken (4)
- Ik ben zeker niet betrokken (5)

1n Contact opnemen met collega's in Duitsland voor afstemming van maatregelen.
- Ik ben zeker betrokken (1)
- Ik ben waarschijnlijk betrokken (2)
- Ik weet het niet (3)
- Ik ben waarschijnlijk niet betrokken (4)
- Ik ben zeker niet betrokken (5)

1o Contact opnemen met collega's in Duitsland voor afstemming van communicatieactiviteiten.

- ❍ Ik ben zeker betrokken (1)
- ❍ Ik ben waarschijnlijk betrokken (2)
- ❍ Ik weet het niet (3)
- ❍ Ik ben waarschijnlijk niet betrokken (4)
- ❍ Ik ben zeker niet betrokken (5)

1p Uw mening is belangrijk voor ons. Heeft u opmerkingen of toevoegingen bij uw antwoorden op het eerste deel van de vragen bij het BRMO-scenario? Deze kunt u hier kwijt:

Dit was de laatste vraag van deel 1. U kunt nu beginnen aan het tweede deel van de vragen op de volgende pagina.

Deel 2. Samenwerking tussen zorgprofessionals
Informatieuitwisseling en samenwerking tussen zorginstellingen en zorgprofessionals is belangrijk waneer een BRMO uitbraak landsgrenzen en instellingen overschrijdt. Daarbij hebben zorginstellingen en zorgprofessionals verschillende rollen bij het verstrekken van informatie.
De volgende 7 stellingen gaan over samenwerking bij de uitbraakbestrijding.
Let op: Wederom, waneer er gesproken wordt van "de uitbraak", wordt daarmee de gehele grens- en instellingsoverschrijdende uibraak, zoals beschreven in het scenario, mee bedoeld.
U kunt het scenario teruglezen door op deze link te klikken.

2a In de bestrijding van deze uitbraak, geef ik waarschijnlijk advies en/of informatie aan de volgende zorginstellingen/zorprofessionals:
U kunt meerdere opties selecteren.

2b In de bestrijding van deze uitbraak, krijg ik waarschijnlijk advies en/of informatie van de volgende zorginstellingen/zorprofessionals: U kunt meerdere opties selecteren.

2c In de bestrijding van deze uitbraak, werk ik waarschijnlijk het meest intensief samen met de volgende zorginstellingen/zorgprofessionals: U kunt meerdere opties selecteren.

2d In de bestrijding van deze uitbraak, ben ik waarschijnlijk afhankelijk van een bijdrage van de volgende zorginstellingen/zorgprofessionals: Met afhankelijk wordt bedoeld, dat u niet verder kunt werken zonder de bijdrage van deze persoon of organisatie of dat de kwaliteit van uw werk significant in het geding komt. Onder een bijdrage verstaan wij bijvoorbeeld: Informatie, materiaal, advies, financiële bijdrage etc. U kunt meerdere opties selecteren.

2e Los van uitbraakbestrijding, werk ik al samen met de volgende zorginstellingen/zorgprofessionals voor andere zorgverlening: Dit wil zeggen: Niet specifiek in de voorbereiding op - of bestrijding van ziekte-uitbraken, maar in uw dagelijkse werkzaamheden. U kunt meerdere opties selecteren.

2f De volgende zorgprofessional(s) zou(den) volgens mij het voortouw moeten nemen in de bestrijding van de uitbraak: Hiermee wordt bedoeld dat de professional(s) een leidende rol aan zou(den) moeten nemen. U kunt ook een zorginstelling in het algemeen selecteren. De zorginstellingen zijn vetgedrukt weergegeven. U kunt meerdere opties selecteren.

2g De volgende zorgprofessional(s) zou(den) volgens mij de samenwerking tussen de verschillende zorginstellingen en zorgprofessionals moeten coördineren tijdens de bestrijding van de uitbraak:Hiermee wordt bedoeld dat de professional(s) een faciliterende rol aan zou(den) moeten nemen. U kunt ook alleen een zorginstelling selecteren. De zorginstellingen zijn vetgedrukt weergegeven. U kunt meerdere opties selecteren.

2h Uw mening is belangrijk voor ons. Heeft u opmerkingen of toevoegingen bij uw antwoorden op het tweede deel van de vragen bij het BRMO-scenario? Deze kunt u hier kwijt:

Dit was de laatste vraag van deel 2. U kunt nu beginnen aan het derde en tevens laatste deel van deze vragenlijst op de volgende pagina.

Deel 3. Stellingen over de uitbraakbestrijding
Tot slot volgen er nu een aantal stellingen over de uitbraakbestrijding. Kunt u voor iedere stelling aangeven in hoeverre u het er mee eens of mee oneens bent?   Let op: Wederom, wanneer er gesproken wordt van "de uitbraak", wordt daarmee de gehele grens-en instellingsoverschrijdende uitbraak, zoals beschreven in het scenario, bedoeld.   U kunt het scenario teruglezen door op deze link te klikken.

3a Bij het invullen van de vragen over activiteiten bij het BRMO scenario, was het voor mij duidelijk bij welke activiteiten ik betrokken zou moeten zijn.

- ❍ Helemaal eens (1)
- ❍ Een beetje mee eens (2)
- ❍ Niet eens en niet oneens (3)
- ❍ Een beetje mee oneens (4)
- ❍ Helemaal mee oneens (5)

3b Het is voor mij duidelijk welke zorginstelling(en)/zorgprofessional(s) het voortouw zou(den) moeten nemen bij de bestrijding van deze uitbraak.

- ❍ Helemaal eens (1)
- ❍ Een beetje mee eens (2)
- ❍ Niet eens en niet oneens (3)
- ❍ Een beetje mee oneens (4)
- ❍ Helemaal mee oneens (5)

3c Het is voor mij duidelijk welke zorginstelling(en)/zorgprofessional(s) een coördinerende rol op zich zou(den) moeten nemen bij de bestrijding van deze uitbraak.
❍ Helemaal eens (1)
❍ Een beetje mee eens (2)
❍ Niet eens en niet oneens (3)
❍ Een beetje mee oneens (4)
❍ Helemaal mee oneens (5)

3d Het is voor mij duidelijk van welke zorgprofessionals ik advies of informatie zal krijgen bij de bestrijding van deze uitbraak.
❍ Helemaal eens (1)
❍ Een beetje mee eens (2)
❍ Niet eens en niet oneens (3)
❍ Een beetje mee oneens (4)
❍ Helemaal mee oneens (5)

3e Het is voor mij duidelijk aan welke zorgprofessionals ik advies of informatie zal geven bij de bestrijding van deze uitbraak.
❍ Helemaal eens (1)
❍ Een beetje mee eens (2)
❍ Niet eens en niet oneens (3)
❍ Een beetje mee oneens (4)
❍ Helemaal mee oneens (5)

3f Het is voor mij duidelijk met welke zorgprofessionals ik het meest intensief zal samenwerken bij de bestrijding van deze uitbraak.
❍ Helemaal eens (1)
❍ Een beetje mee eens (2)
❍ Niet eens en niet oneens (3)
❍ Een beetje mee oneens (4)
❍ Helemaal mee oneens (5)

3g Ik denk dat ik voldoende capaciteiten heb om juist te handelen bij de bestrijding van deze uitbraak.
❍ Helemaal eens (1)
❍ Een beetje mee eens (2)
❍ Niet eens en niet oneens (3)
❍ Een beetje mee oneens (4)
❍ Helemaal mee oneens (5)

3h Ik denk dat de andere betrokken zorgprofessionals voldoende capaciteiten hebben om juist te handelen bij de bestrijding van deze uitbraak.
❍ Helemaal eens (1)
❍ Een beetje mee eens (2)
❍ Niet eens en niet oneens (3)
❍ Een beetje mee oneens (4)
❍ Helemaal mee oneens (5)

3i Ik denk dat de betrokken zorgprofessionals het collectief belang van de uitbraakbestrijding voor het eigen-/instellingsbelang laten gaan.
❍ Helemaal eens (1)
❍ Een beetje mee eens (2)
❍ Niet eens en niet oneens (3)
❍ Een beetje mee oneens (4)
❍ Helemaal mee oneens (5)

3j Ik denk dat de betrokken zorgprofessionals dezelfde ideeën hebben als ik over een juiste bestrijding van deze uitbraak.
❍ Helemaal eens (1)
❍ Een beetje mee eens (2)
❍ Niet eens en niet oneens (3)
❍ Een beetje mee oneens (4)
❍ Helemaal mee oneens (5)

3k Ik zou graag meer duidelijkheid hebben over de rollen en verantwoordelijkheden van zorginstellingen en zorgprofessionals bij de bestrijding van grens- en instellingsoverschijdende BRMO uitbraken.
❍ Helemaal eens (1)
❍ Een beetje mee eens (2)
❍ Niet eens en niet oneens (3)
❍ Een beetje mee oneens (4)
❍ Helemaal mee oneens (5)

3l Heeft u opmerkingen of toevoegingen bij uw antwoorden op het derde deel van de vragen bij het BRMO-scenario? Deze kunt u hier kwijt:

Dit was de laatste vraag van deel 3. Op de volgende pagina vindt u de afsluiting van de vragenlijst.

Dit is het einde van de vragenlijst. Wanneer u uw antwoorden nog wilt controleren of veranderen, kunt u hieronder op 'terug' klikken. Uw antwoorden zullen dan niet verloren gaan. Uw mening is belangrijk voor ons. Heeft u nog overige opmerkingen met betrekking tot uw antwoorden in de vragenlijst, of opmerkingen over het onderzoek of de vragenlijst zelf? Deze kunt u hier vermelden:

Wij willen u hartelijk bedanken voor uw bijdrage aan dit onderzoek. Voor verdere vragen over de vragenlijst of het onderzoek kunt u contact opnemen met Jacklien Maessen door te e-mailen naar jacklien.maessen@rivm.nl Vergeet niet om op de blauwe knop ">>" te drukken zodat uw antwoorden verzonden worden.
