# Supplementary material for: How prepared are we for cross-border outbreaks? An exploratory analysis of cross-border response networks for outbreaks of multidrug resistant microorganisms in the Netherlands and Germany
Source: PLoS One. 2019 Jul 10;14(7):e0219548. doi: 10.1371/journal.pone.0219548 (PMC6619808; doi:10.1371/journal.pone.0219548)
Supplement: S2 File — (PDF) [file pone.0219548.s002.pdf]

Rollen und Erwartungen von Stakeholdern bei der Bekämpfung grenzübergreifender Ausbrüche von MRE

Sehr geehrte(r) Herr/Frau,

Herzlichen Dank für Ihre Teilnahme an der Studie Rollen und Erwartungen von Stakeholdern bei der Bekämpfung grenzübergreifender Ausbrüche von multiresistenten Erregern. Dies ist eine Studie der Landelijke coördinatie Infectieziektebestrijding (LCI) (zu Deutsch: Landesweite Koordinierung Bekämpfung von Infektionskrankheiten) des RIVM (zu Deutsch: Niederländisches Institut für Volksgesundheit und Umwelt) in Zusammenarbeit mit Tilburg University.

Mithilfe dieses Fragebogens möchten wir mehr Einsicht erhalten in die unterschiedlichen Rollen und Erwartungen beteiligter Gesundheitsorganisationen bzw. Pflegeeinrichtungen und Pflegedienstleister in Zeiten eines grenz- und einrichtungsübergreifenden Ausbruchs multiresistenten Erregern (MRE). Ziel dieser Studie ist es, das Interventionsnetzwerk zu ermitteln, das im Falle eines grenz- und einrichtungsübergreifenden Ausbruchs von MRE in den Niederlanden und Deutschland mutmasslich in Aktion tritt.

Ihre Teilnahme ist uns sehr wichtig. Die Ergebnisse werden einen Beitrag zur Entwicklung von Richtlinien in Bezug auf die BRMO-Ausbruchsbekämpfung liefern. Das Ausfüllen des Fragebogens dauert ungefähr 15 Minuten. Es ist möglich, Ihre Antworten vorübergehend zu speichern und das Ausfüllen des Fragebogens zu einem späteren Zeitpunkt fortzusetzen. Wie Sie dem Einwilligungsformular auf der nächsten Seite entnehmen können, werden Ihre Antworten vertraulich behandelt und ist Ihre Teilnahme freiwillig.

Für Fragen zur Studie können Sie Kontakt aufnehmen mit Jacklien Maessen, indem Sie eine E-Mail schicken an jacklien.maessen@rivm.nl

Einwilligungsformular

Über diesen Link können Sie die Einwilligungserklärung ansehen.

Hiermit erkläre ich, die Einwilligungserklärung gelesen und verstanden zu haben. Ich verstehe das Ziel der Studie und verstehe, was von mir verlangt wird. Ich verstehe, dass ich meine Teilnahme an dieser Studie jederzeit beenden und beschließen kann, bestimmte Fragen nicht zu beantworten. Ich verstehe, dass eine Teilnahme vertraulich ist und keine individuellen Schlussfolgerungen anhand meines individuellen Beitrages gezogen werden. Ich verstehe, dass die Daten dieser Studie gemäß Protokoll zehn Jahre lang in einer gesicherten Akte aufbewahrt werden. Hiermit erkläre ich, die Möglichkeit gehabt zu haben, Fragen zu stellen und freiwillig an dieser Studie teilzunehmen.

Hiermit erkläre ich unter den oben aufgeführten Bedingungen an der Studie teilzunehmen.
❑ Name des/der Teilnehmers/Teilnehmerin: (1) ____________________

Allgemeine Daten
Es folgen nun 2 allgemeine Fragen über Sie und die Gesundheitsorganisation, in der Sie tätig sind.
In welcher Region sind Sie tätig?
❍ Gesundheitsamt Kreis X (1)
❍ Gesundheitsamt Städteregion Aachen (2)

Wo sind Sie beschäftigt und was ist Ihre Function?
Sind Sie bei mehreren Gesundheitsorganisationen bzw. Pflegeeinrichtungen tätig? Bitte kreuzen Sie dann die Einrichtung an, für die Sie für diese Untersuchung angeschrieben wurden.

Das grenz- und organisationsüberschreitende MRE Ausbruchsszenario
Untenstehend finden Sie eine fiktives Szenario, worin ein grenzüberschreitendes Auftreten von multiresistenten Erregern in den Niederlanden und Deutschland beschrieben wird. Auf Basis dieses Szenarios möchten wir Sie bitten die darauf folgenden Fragen zu beantworten.

Wir bitten Sie darum erst das Szenario sorgfältig durchzulesen.

Seit ein paar Tagen liegt Frau Schmidt (84 Jahre alt, wohnhaft in Kalkar) auf Grund von andauerndem hohem Fieber und niedrigem Blutdrucks auf der 'ambulanten Station' in einem Krankenhaus in der Region X. Der behandelnde Arzt hat festgestellt, dass die Ursache wahrscheinlich eine Urosepsis ist.

Das Antibiotikum, welches zur Behandlung eingesetzt wurde (Cephalosporin der dritten Generation), zeigt keine Wirkung. Das Medizinische Mikrobiologie Labor stellt nach zwei Tagen Kultivierung fest, dass sich im Urin von Frau Schmidt NDM-1 produzierende Klebsiella pneumoniae befindet. Diese Art von Klebsiella pneumoniae ist resistent gegen co-trimoxazol und fluorchinolonen, aber empfindlich gegenüber aminoglycosiden, tigecycline und colistine.

Es wird eine neue Anamnese durchgeführt. Es scheint, dass Frau Schmidt vor kurzem mit ähnlichen Symptomen in einem Krankenhaus in Griechenland aufgenommen wurde, dies aber bei der Aufnahme nicht angegeben hat. Nach Abklingen der Symptome in Griechenland flog sie nach Deutschland zurück. Wieder zuhause, wurde Sie vier Tage später im Krankenhaus Region X aufgenommen.

Frau Schmidt wird, gemäß Protokoll, von nun an isoliert behandelt. Bei ihren vorherigen Zimmergenossen werden Kulturproben genommen um zu sehen ob diese Träger von NDM-1 produzierender Klebsiella pneumoniae sind (Ermittlung von Kontaktpersonen/'erster Ring' Kontakt Untersuchung).

Das Ergebnis dieser ersten Kontaktuntersuchung ist Anlass um den Ring der Kontaktuntersuchung auf die Krankenhausabteilung zu vergrößern. Basierend auf den Ergebnissen dieser zweiten Kontaktuntersuchung wird entschieden alle (ehemaligen) Patienten der Abteilung, vom Zeitpunkt der Aufnahme von Frau Schmidt, zu screenen.

Insgesamt wurden 38 (ehemalige) Patienten gescreent auf Trägerschaft von NDM-1 produzierende Klebsiella pneumoniae. Zehn der gescreenten Patienten wurden positiv getestet. Zwei dieser Patienten befinden sich noch im Krankenhaus in der Region X. Drei ehemalige Patienten mit Trägerschaft wohnen in der Region X. Basierend auf langjährigen gesundheitlichen Problemen, besuchen diese Menschen regelmäßig das Krankenhaus und sind alle drei zusätzlich abhängig von häuslicher Pflege. Ein anderer positiv getesteter ehemaliger Patient befindet sich in ein Pflegeheim in der Region X.

Zwei von den positiv getesteten ehemalige Patienten wurden bereits in ein Pflegeheim in der Niederländischen Region X verlegt. Eine Kontaktuntersuchung in diesem Pflegeheim ergab einen positiven Bewohner, welcher aufgrund anderer gesundheitlichen Beschwerden inzwischen im Krankenhaus aufgenommen wurde. Ein anderer MRE-träger hat seinen Wohnsitz in der Region X. Auch er ist aufgrund Nebenerkrankungen abhängig von häuslicher Versorgung und besucht regelmässig das Krankenhaus.

Heute Morgen war in einer Niederländischen Zeitung die folgende Schlagzeile zu lesen: "Deutsches Krankenhaus verursacht Ausbruch Superbakterie in den Niederlanden". Die Schlagzeile einer Deutschen Zeitung lautete: "Ausbruch von Superbakterie in der Region X". Beide Artikel beschreiben die persönliche Geschichte von eines der Patienten und lassen sich über das wachsende Risiko von multiresistente Erregern aus.

Sie können das Szenario in diesem Fragebogen jederzeit erneut lesen, indem Sie auf den folgenden Link klicken: Link
Über diesen Link sehen Sie auch ein Diagramm, dass Ihnen eine schematische Übersicht des Szenarios vermittelt.
Auf der folgenden Seite finden Sie eine kurze Einleitung in die Fragen dieses Fragebogens.

Einleitung in die Fragen basierend auf dem fiktiven MRE-szenario
Im folgendem stellen wir Ihnen in drei Teilen Fragen über das MRE Ausbruch Szenario:
Teil 1 geht über grenz- und Institution bzw. organisationsübergreifende Aktivitäten.
Teil 2 geht über die Zusammenarbeit von medizinischen Fachkräften.
Teil 3 besteht aus einer Anzahl von Aussagen über die Ausbruchsbekämpfung.

Wir bitten Sie, jede Frage aus Perspektive Ihrer eigenen Funktion in der Organisation zu beantworten.

Weiterhin ist es bei der Beantwortung der Fragen wichtig davon auszugehen, dass Sie Gesundheitsinstitutionen oder medizinisches Fachpersonal, benannt im Szenario, auf sich selbst beziehen. Innerhalb Ihres Teams sind Sie Ansprechpunkt bei diesem Ausbruch. Zum Beispiel, Sie sind Arzt in einem Pflegeheim und im Szenario ist die Rede von einem Pflegeheim, dann können Sie davon ausgehen, dass dies Ihr Pflegeheim ist und Sie hier der verantwortliche Arzt in dem Szenario sind. Das gleiche gilt wenn Sie, zum Beispiel, Hausarzt sind und es ist die Rede von Menschen in der häuslichen Situation; Sie können dann davon ausgehen, dass Sie der Hausarzt eines oder mehrerer dieser Patienten sind.

Sie können nun mit dem ersten Teil dieses Fragebogens auf der folgenden Seite beginnen.

Teil 1. Grenz- und Institutionsübergreifende Aktivitäten
Im folgenden sind 15 Aktivitäten benannt, die bei einem grenz- und institutionsübergreifendem MRE Ausbruch stattfinden können. Basierend auf dem Szenario, können Sie für jede dieser Tätigkeiten angeben, wie wahrscheinlich es ist, dass Sie hieran beteiligt sind?

Bitte Beachten Sie, das mit " dieser Ausbruch" der vollständige grenz- und einrichtungsübergreifende Ausbruch gemeint is, wie im Szenario beschrieben. Sie können das Szenario nochmals lesen, indem Sie auf diesen Link klicken.

1a Teilnahme an einem Ausbruch Management-Team, um über Maßnahmen zu entscheiden um den Ausbruch zu bekämpfen.
❍ Ich bin sicherlich beteiligt (1)
❍ Ich bin wahrscheinlich beteiligt (2)
❍ Ich weiß es nicht (3)
❍ Ich bin wahrscheinlich nicht beteiligt (4)
❍ Ich bin sicherlich nicht beteiligt (5)

1b Screening von ehemaligen Zimmergenossen von Frau Schmidt, die sich außerhalb des Krankenhauses befinden (im Pflegeheim oder zu Hause).
❍ Ich bin sicherlich beteiligt (1)
❍ Ich bin wahrscheinlich beteiligt (2)
❍ Ich weiß es nicht (3)
❍ Ich bin wahrscheinlich nicht beteiligt (4)
❍ Ich bin sicherlich nicht beteiligt (5)

1c Ausweitung von Maßnahmen zur Infektionsprävention (implementieren von zusätzlichen Maßnahmen) im Pflegeheim.
❍ Ich bin sicherlich beteiligt (1)
❍ Ich bin wahrscheinlich beteiligt (2)
❍ Ich weiß es nicht (3)
❍ Ich bin wahrscheinlich nicht beteiligt (4)
❍ Ich bin sicherlich nicht beteiligt (5)

1d Implementieren von Maßnahmen zur Infektionsprävention in der häuslichen Situation von MRE positiv befundenen Personen (Schutzmaßnahmen bei körperlichen Pflege).
❍ Ich bin sicherlich beteiligt (1)
❍ Ich bin wahrscheinlich beteiligt (2)
❍ Ich weiß es nicht (3)
❍ Ich bin wahrscheinlich nicht beteiligt (4)
❍ Ich bin sicherlich nicht beteiligt (5)

1e Bereitstellen von Informationen an MRE positiv befundene Personen in der häuslichen Situation.
❍ Ich bin sicherlich beteiligt (1)
❍ Ich bin wahrscheinlich beteiligt (2)
❍ Ich weiß es nicht (3)
❍ Ich bin wahrscheinlich nicht beteiligt (4)
❍ Ich bin sicherlich nicht beteiligt (5)

1f Beantworten von Fragen aus der Öffentlichkeit über den Ausbruch (hierbei sind nicht die Personen gemeint, die bei Kontaktuntersuchungen betroffen waren).
❍ Ich bin sicherlich beteiligt (1)
❍ Ich bin wahrscheinlich beteiligt (2)
❍ Ich weiß es nicht (3)
❍ Ich bin wahrscheinlich nicht beteiligt (4)
❍ Ich bin sicherlich nicht beteiligt (5)

1g Austausch von Patientendaten zwischen medizinischen Fachkräften und Institutionen des Gesundheitswesens zum Zwecke der Ausbruchsbekämpfung.
❍ Ich bin sicherlich beteiligt (1)
❍ Ich bin wahrscheinlich beteiligt (2)
❍ Ich weiß es nicht (3)
❍ Ich bin wahrscheinlich nicht beteiligt (4)
❍ Ich bin sicherlich nicht beteiligt (5)

1h Kommunikation mit den Medien über den Ausbruch.
❍ Ich bin sicherlich beteiligt (1)
❍ Ich bin wahrscheinlich beteiligt (2)
❍ Ich weiß es nicht (3)
❍ Ich bin wahrscheinlich nicht beteiligt (4)
❍ Ich bin sicherlich nicht beteiligt (5)

1i Aufrechterhalten von / Beitragen an einem Grenz- und Institutionsübergreifendes Fallregister des Ausbruchs.
❍ Ich bin sicherlich beteiligt (1)
❍ Ich bin wahrscheinlich beteiligt (2)
❍ Ich weiß es nicht (3)
❍ Ich bin wahrscheinlich nicht beteiligt (4)
❍ Ich bin sicherlich nicht beteiligt (5)

1j Evaluieren von grenz- und institutionsübergreifender Zusammenarbeit und den getroffenen Kontrollmaßnahmen.
❍ Ich bin sicherlich beteiligt (1)
❍ Ich bin wahrscheinlich beteiligt (2)
❍ Ich weiß es nicht (3)
❍ Ich bin wahrscheinlich nicht beteiligt (4)
❍ Ich bin sicherlich nicht beteiligt (5)

1k Informieren von öffentlichen Amtsträgern (Bürgermeister, Stadträte, Regierungspräsident, etc.) über den Ausbruch.
❍ Ich bin sicherlich beteiligt (1)
❍ Ich bin wahrscheinlich beteiligt (2)
❍ Ich weiß es nicht (3)
❍ Ich bin wahrscheinlich nicht beteiligt (4)
❍ Ich bin sicherlich nicht beteiligt (5)

1l Kontaktaufnahme mit Kollegen/innen in den Niederlanden zum Zwecke des Austauschs von Informationen.
❍ Ich bin sicherlich beteiligt (1)
❍ Ich bin wahrscheinlich beteiligt (2)
❍ Ich weiß es nicht (3)
❍ Ich bin wahrscheinlich nicht beteiligt (4)
❍ Ich bin sicherlich nicht beteiligt (5)

1m Kontaktaufnahme mit Kollegen/innen in den Niederlanden zum Zwecke der Abstimmung von Massnahmen.
❍ Ich bin sicherlich beteiligt (1)
❍ Ich bin wahrscheinlich beteiligt (2)
❍ Ich weiß es nicht (3)
❍ Ich bin wahrscheinlich nicht beteiligt (4)
❍ Ich bin sicherlich nicht beteiligt (5)

1n Kontaktaufnahme mit Kollegen/innen in den Niederlanden zum Zwecke der Abstimmung der Kommunikationsstrategie nach aussen.
❍ Ich bin sicherlich beteiligt (1)
❍ Ich bin wahrscheinlich beteiligt (2)
❍ Ich weiß es nicht (3)
❍ Ich bin wahrscheinlich nicht beteiligt (4)
❍ Ich bin sicherlich nicht beteiligt (5)

1o Meldung des grenz-und institutionsüberschreitenden Ausbruchs an die regionalen öffentlichen Autoritäten.

- ❍ Ich bin sicherlich beteiligt (1)
- ❍ Ich bin wahrscheinlich beteiligt (2)
- ❍ Ich weiß es nicht (3)
- ❍ Ich bin wahrscheinlich nicht beteiligt (4)
- ❍ Ich bin sicherlich nicht beteiligt (5)

1p Haben Sie noch Kommentare oder Ergänzungen zu Ihren Antworten auf diesen ersten Teil der Fragen zum MRE Szenario? Diese können Sie hier eintragen:

Das war die letzte Frage von Teil 1. Sie können nun mit dem zweiten Teil des Fragebogens auf der folgenden Seite beginnen.

Teil 2. Zusammenarbeit zwischen Gesundheitsorganisationen/Pflegedienstleistern

Informationsaustausch und Zusammenarbeit zwischen Pflegeeinrichtungen und Pflegedienstleistern ist wichtig, wenn ein Ausbruch von MRE Landesgrenzen und Einrichtungen überschreitet. Dabei nehmen Pflegeeinrichtungen und Pflegedienstleister verschiedene Rollen in Bezug auf die Bereitstellung von Informationen ein.

Die folgenden 7 Aussagen beziehen sich auf die Zusammenarbeit bei der Ausbruchsbekämpfung.
Bitte Beachten Sie, das mit " dieser Ausbruch" der vollständige grenz- und einrichtungsübergreifende Ausbruch gemeint is, wie im Szenario beschrieben.
Sie können das Szenario nochmals lesen, indem Sie auf diesen Link klicken.

2a Bei der Bekämpfung dieses Ausbruchs berate oder informiere ich wahrscheinlich die folgenden Gesundheitsorganisationen/Pflegeeinrichtungen/Pflegedienstleister:

2b Bei der Bekämpfung dieses Ausbruchs beraten oder informieren mich wahrscheinlich die folgenden Gesundheitsorganisationen/Pflegeeinrichtungen/Pflegedienstleister: Sie können mehrere Optionen auswählen.

2c Bei der Bekämpfung dieses Ausbruchs arbeite ich wahrscheinlich am intensivsten mit folgenden Gesundheitsorganisationen/Pflegeeinrichtungen/Pflegedienstleistern zusammen: Sie können mehrere Optionen auswählen.

2d Bei der Bekämpfung dieses Ausbruchs bin ich wahrscheinlich abhängig von einem Beitrag der folgenden Gesundheitsorganisationen/Pflegeeinrichtungen/Pflegedienstleister:
Mit abhängig wird gemeint, dass Sie ohne den Beitrag dieser Person oder Organisation nicht weiterarbeiten können, oder dass die Qualität Ihrer Arbeit signifikant gefährdet wird.
Ein Beitrag ist in diesem Sinne zum Beispiel: Information, Material, Beratung, finanzieller Beitrag, etc. Sie können mehrere Optionen auswählen.

2e Unabhängig von der Ausbruchsbekämpfung arbeite ich auf dem Gebiet anderer Pflegedienstleistungen bereits mit den folgenden Gesundheitsorganisationen/Pflegeeinrichtungen/Pflegedienstleistern zusammen:
Das heißt: Nicht speziell auf dem Gebiet der Vorbereitung auf - oder Bekämpfung von - Krankheitsausbrüchen, sondern während Ihrer täglichen Tätigkeiten. Sie können mehrere Optionen auswählen.

2f Der/die folgende(n) Gesundheitsorganisation bzw. Pflegedienstleister müsste(n) meiner Meinung nach eine führende Rolle bei der Bekämpfung des Ausbruchs einnehmen:
Sie können auch lediglich eine Gesundheitsorganization/Pflegeeinrichtung wählen. Die Gesundheitsorganisation/Pflegeeinrichtungen werden fettgedruckt angezeigt.
Sie können mehrere Optionen auswählen.

2g Der/die folgende(n) Gesundheitsorganisationen bzw. Pflegedienstleister müsste(n) meiner Meinung nach die Zusammenarbeit zwischen den verschiedenen Gesundheitsorganizationen, Pflegeeinrichtungen und Pflegedienstleistern während der Bekämpfung des Ausbruchs koordinieren:
Sie können auch lediglich eine Gesundheitsorganisation bzw. Pflegeeinrichtung wählen. Die Gesundheitsorganisationen/Pflegeeinrichtungen werden fettgedruckt angezeigt.
Sie können mehrere Optionen auswählen.

2h Haben Sie Anmerkungen oder Ergänzungen zu Ihren Antworten auf den zweiten Teil der Fragen beim MRE-Szenario? Diese können Sie hier eintragen:

Dies war die letzte Frage von Teil 2. Sie können nun auf der nächsten Seite mit dem dritten und letzten Teil dieses Fragebogens beginnen.

Teil 3. Aussagen über die Ausbruchsbekämpfung
Zum Schluss folgt nun eine Reihe von Aussagen über die Ausbruchsbekämpfung. Wir bitten Sie, für jede Aussage anzugeben, inwiefern Sie dieser zustimmen oder nicht zustimmen.
Bitte Beachten Sie, das mit " dieser Ausbruch" der vollständige grenz- und einrichtungsübergreifende Ausbruch gemeint is, wie im Szenario beschrieben.
Sie können das Szenario nochmals lesen, indem Sie auf diesen Link klicken.

3a Beim Ausfüllen der Fragen zu den Aktivitäten beim MRE-Szenario war mir klar, an welchen Aktivitäten ich mich beteiligen müsste.
❍ Stimme voll und ganz zu (1)
❍ Stimme zu (2)
❍ Neutral (3)
❍ Stimme nicht zu (4)
❍ Stimme überhaupt nicht zu (5)

3b Mir ist klar, welche(r) Gesundheitsorganisation(en)/Pflegeeinrichtung(en)/Pflegedienstleister eine führende Rolle bei der Bekämpfung dieses Ausbruchs einnehmen sollte(n).
❍ Stimme voll und ganz zu (1)
❍ Stimme zu (2)
❍ Neutral (3)
❍ Stimme nicht zu (4)
❍ Stimme überhaupt nicht zu (5)

3c Mir ist klar, welche(r) Gesundheitsorganisation(en)/Pflegeeinrichtung(en)/Pflegedienstleister eine koordinierende Rolle bei der Bekämpfung dieses Ausbruchs einnehmen sollte(n).
❍ Stimme voll und ganz zu (1)
❍ Stimme zu (2)
❍ Neutral (3)
❍ Stimme nicht zu (4)
❍ Stimme überhaupt nicht zu (5)

3d Mir ist klar, von welchen Gesundheitsorganisationen bzw. Pflegedienstleistern ich bei der Bekämpfung dieses Ausbruchs Ratschläge oder Informationen erhalte.
❍ Stimme voll und ganz zu (1)
❍ Stimme zu (2)
❍ Neutral (3)
❍ Stimme nicht zu (4)
❍ Stimme überhaupt nicht zu (5)

3e Mir ist klar, welchen Gesundheitsorganisationen bzw. Pflegedienstleistern ich bei der Bekämpfung dieses Ausbruchs Ratschläge oder Informationen liefern kann.
❍ Stimme voll und ganz zu (1)
❍ Stimme zu (2)
❍ Neutral (3)
❍ Stimme nicht zu (4)
❍ Stimme überhaupt nicht zu (5)

3f Mir ist klar, mit welchen Gesundheitsorganisationen bzw. Pflegedienstleistern ich bei der Bekämpfung dieses Ausbruchs am intensivsten zusammenarbeiten werde.
❍ Stimme voll und ganz zu (1)
❍ Stimme zu (2)
❍ Neutral (3)
❍ Stimme nicht zu (4)
❍ Stimme überhaupt nicht zu (5)

3g Ich denke, dass ich über genügend Kenntnisse und Fähigkeiten verfüge, um bei der Bekämpfung dieses Ausbruchs korrekt zu handeln.
❍ Stimme voll und ganz zu (1)
❍ Stimme zu (2)
❍ Neutral (3)
❍ Stimme nicht zu (4)
❍ Stimme überhaupt nicht zu (5)

3h Ich denke, dass andere beteiligte Gesundheitsorganisationen bzw. Pflegedienstleister über genügend Kenntnisse und Fähigkeiten verfügen, um bei der Bekämpfung dieses Ausbruchs korrekt zu handeln.
❍ Stimme voll und ganz zu (1)
❍ Stimme zu (2)
❍ Neutral (3)
❍ Stimme nicht zu (4)
❍ Stimme überhaupt nicht zu (5)

3i An der Bekämpfung dieses Ausbruchs sind Gesundheitsorganisationen und Pflegedienstleister beteiligt, mit denen ich lieber nicht zusammenarbeite.
❍ Stimme voll und ganz zu (1)
❍ Stimme zu (2)
❍ Neutral (3)
❍ Stimme nicht zu (4)
❍ Stimme überhaupt nicht zu (5)

3j Ich denke, dass das allgemeine Interesse auf dem Gebiet der Ausbruchsbekämpfung für die beteiligten Gesundheitsorganisationen und Pflegedienstleister schwerer wiegt als das eigene Interesse/Interesse der Einrichtung.
- ❍ Stimme voll und ganz zu (1)
- ❍ Stimme zu (2)
- ❍ Neutral (3)
- ❍ Stimme nicht zu (4)
- ❍ Stimme überhaupt nicht zu (5)

3k Ich denke, dass ich und die beteiligten Gesundheitsorganisationen und Pflegedienstleister bei der Bekämpfung dieses Ausbruchs dieselben Ideen vertreten.
- ❍ Stimme voll und ganz zu (1)
- ❍ Stimme zu (2)
- ❍ Neutral (3)
- ❍ Stimme nicht zu (4)
- ❍ Stimme überhaupt nicht zu (5)

3l Ich wünsche mir gerne mehr Klarheit über die Rollen und Verantwortlichkeiten von Gesundheitsorganisationen und Pflegeeinrichtungen und Pflegedienstleistern bei der Bekämpfung von grenz- und einrichtungsübergreifenden BRMO-Ausbrüchen.
- ❍ Stimme voll und ganz zu (1)
- ❍ Stimme zu (2)
- ❍ Neutral (3)
- ❍ Stimme nicht zu (4)
- ❍ Stimme überhaupt nicht zu (5)

Haben Sie Anmerkungen oder Ergänzungen zu Ihren Antworten auf den dritte Teil der Fragen beim MRE-Szenario? Diese können Sie hier eintragen:

Dies war die letzte Frage von Teil 3. Auf der nächsten Seite finden Sie den abschließ dieses Fragebogens.

Dies ist das Ende des Fragebogens. Falls Sie Ihre Antworten noch kontrollieren oder ändern möchten, können Sie hier unten auf „Zurück“ klicken. Ihre Antworten gehen dann nicht verloren.
Haben Sie noch weitere Anmerkungen in Bezug auf Ihre Antworten im Fragebogen oder Anmerkungen zum Fragebogen selbst? Diese können Sie hier aufschreiben:

Wir möchten Ihnen herzlich für Ihren Beitrag zu dieser Studie danken.
Für weitere Fragen zum Fragebogen oder zur Studie können Sie Kontakt aufnehmen mit Jacklien Maessen, indem Sie eine E-Mail schicken an jacklien.maessen@rivm.nl
Vergessen Sie nicht, auf den blauen Knopf ">>“ zu klicken, sodass Ihre Antworten verschickt werden.
