# Supplementary material for: How prepared are we for cross-border outbreaks? An exploratory analysis of cross-border response networks for outbreaks of multidrug resistant microorganisms in the Netherlands and Germany
Source: PLoS One. 2019 Jul 10;14(7):e0219548. doi: 10.1371/journal.pone.0219548 (PMC6619808; doi:10.1371/journal.pone.0219548)
Supplement: S3 File — (PDF) [file pone.0219548.s003.pdf]

Roles and expectations in the containment of cross-border outbreaks of MDRO

It must be noted that this questionnaire is translated from Dutch to English for the pure purpose of publication in the online data repository and that it is not distributed in English. The German version of this questionnaire is translated from Dutch by professional translators and is distributed in that form.

Dear Sir/Madam,

Thank you for your participation in the research 'Roles and expectations of stakeholders in the containment of cross-border outbreaks of multidrug resistant micro-organisms'. This is a study of the Dutch National Coordination Centre of Infectious Disease Control (LCI) which is part of the National Institute of Public Health and the Environment (RIVM) in cooperation with Tilburg University.

With this questionnaire we want to gain more insight into the different roles and expectations of involved healthcare institutions and health care professionals, at the time of a cross-border outbreak of multidrug resistant micro-organisms (MDRO). The aim of this research is to map out a response network that comes into effect in the event of a cross-border outbreak of MDRO in the Netherlands and Germany.

Your participation is important to us. The results will contribute to the development of guidelines for MDRO outbreak control. Filling in the questionnaire takes about 15 minutes. It is possible to save your answers in the interim and to resume filling in the questionnaire at a later date. As you can read in the consent form on the next page, your answers will be treated confidentially and your participation is voluntary.

For questions about the research, please contact Jacklien Maessen by e-mailing jacklien.maessen@rivm.nl

Permission form

Via this link you can view the permission statement.

With this I indicate that I have read and understood the declaration of consent. I understand the purpose of the research and understand what is asked of me. I understand that I can stop my participation in this survey at any time and that I can decide not to answer questions. I understand that participation is confidential and that no conclusions are drawn on the basis of my individual contribution. I understand that the data from this research according to the protocol will be kept for ten years in a secure file. I hereby state that I have had the opportunity to ask questions and that I voluntarily participate in this research.

With this I agree with the declaration of consent:
○ I agree with the declaration of consent (1) ____________________

General data

There are now 2 general questions about you and the care institution in which you work.

In which region are you working?
- GGD region (1)
- GGD region (2)

Where are you working and what is your job?
Are you working in several healthcare institutions? Then select the healthcare institution that you have contacted for participation in this study.

- Hospital - Board of Directors / Management (1)
- Hospital - Expert Infection Prevention (2)
- Hospital - Doctor Microbiologist (3)
- Hospital - Infectiologist (4)

- Hospital - Department Manager (5)
- Hospital - Communication Adviser (7)
- Public Health Service - Public Health Director (8)
- GGD - Expert Infection Prevention (9)
- GGD - Infectious disease control doctor (10)
- GGD / Gesundheitsamt - Nurse (11)
- GGD - Communication Adviser (12)
- Home care - Board of Directors / Management (13)
- Home care - Nurse / Carer (14)
- Home care - Communication Advisor (15)
- Nursing home - Board of Directors / Management (16)
- Nursing Home - Expert Infection Prevention (17)
- Nursing Home - Specialist in Geriatric Medicine (18)
- Nursing home - Communication Adviser (19)
- Regional Laboratory of Medical Microbiology - Physician-Microbiologist (20)
- Region Network - Coordinator / Quartermaker (21)
- RIVM - LCI (22)
- Other, namely: (23) ____________________

The cross-border and cross-institution MDRO outbreak scenario

Below is a fictitious scenario describing a cross-border outbreak of multidrug resistant microorganisms in both the Netherlands and Germany. Based on this scenario, we will ask you a number of questions.

We request that you carefully read this scenario.

Since a few days Mrs Schmidt (84 years and living in the German X) in a hospital in the X region in the department 'short-term admissions' in connection with persistent high fever and low blood pressure. The treating physician finds that there is urosepsis.

The antibiotic administered (third generation cephalosporin) does not affect. After two days of culture, the microbiology laboratory shows an infection with a NDM-1 producing Klebsiella pneumoniae in the urine of Ms. Schmidt. This type of Klebsiella pneumoniae is resistant to co-trimoxazole and fluoroquinolones, but sensitive to aminoglycosides, tigecycline and colistin.

A new anamnesis is performed. This shows that Ms. Schmidt has recently been admitted to hospital in Greece with similar complaints, but has not told you about admission. She returned to Germany after improvement of the complaints. After being at home for four days she was admitted to the hospital.

In accordance with the protocol, Mrs Schmidt will now be treated in isolation. In her current roommates, cultures are taken to see if they are carriers of this NDM-1 producing Klebsiella pneumoniae (ring / contact examination).

The outcome of this first ring test is the reason to carry out a larger ring test at the hospital department. Based on the outcome of this follow-up investigation in the department, it is decided to screen all (former) patients of the department of Ms. Schmidt since her admission.

A total of 38 (ex-) patients are screened for carrier status of this NDM-1 producing Klebsiella pneumoniae. Ten of the screened (ex) patients are found positive. Two of these patients are still in hospital in the X region. Three of the former patients with carrier status are at home in the X region. These people visit the hospital regularly because of their longer-term health problems and all three also depend on home care for their daily care. One ex-patient found positive is in a nursing home in the X region.

Two of the positive found former patients were transferred to a nursing home in the nearby Dutch region of X. A follow-up research by the nursing home shows that one other resident has been found to be positive and has since been admitted to a hospital in X in connection with other health problems. In addition, one ex-patient with carrier status is at home in the X region. This person also suffers from underlying suffering and frequently visits the hospital. This ex-patient is also dependent on home care.

This morning, the regional Dutch newspaper headlines "German hospitalized outbreak of super bacteria in the Netherlands". The German newspaper headlines "Outbreak of super bacteria in the X region". Both articles describe the personal account of one of the patients and meadows about the growing risks of resistant micro-organisms.

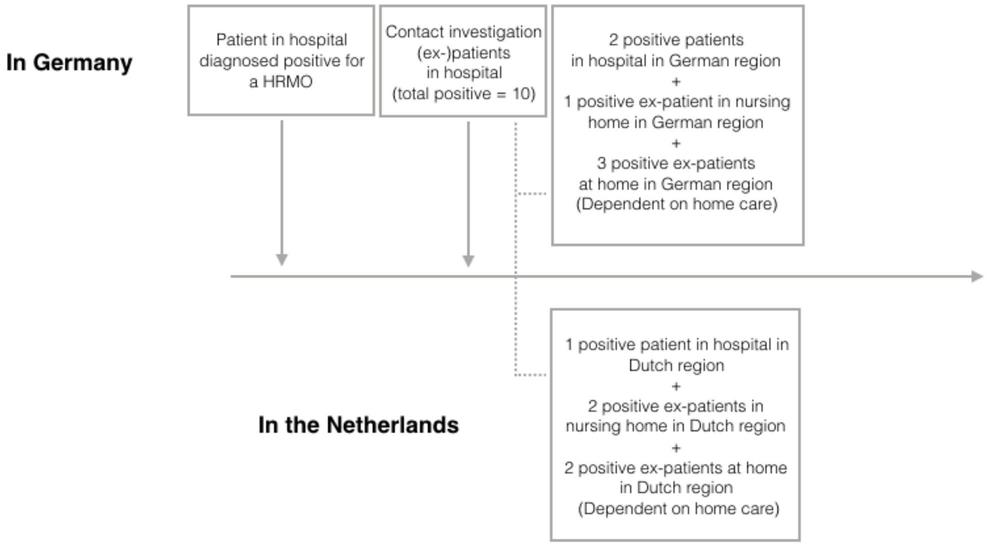

Later in this questionnaire, you can still reread the scenario including the schematic overview by clicking on this link.

Introduction of questions about the fictitious cross-border MDRO scenario

We will ask you in three parts questions about the cross-border MDRO outbreak scenario:
Part 1 deals with control activities.
Part 2 is about cooperation between healthcare professionals.
Part 3 consists of a number of concluding statements about outbreak control.

We ask you to answer every question from your own position in your organization. Furthermore, when answering the questions, it is important to assume that the healthcare institutions or healthcare professionals mentioned in the scenario are related to you. You are the contact person for this outbreak within your team of colleagues. For example, you are a doctor in a Dutch nursing home and there is talk of a Dutch nursing home in the scenario; You can then make the assumption that it is your nursing home that is involved in this scenario and that you are the responsible physician.

You can now start with the first part of the questions on the next page.

Part 1. Cross-border and cross-institution outbreak activities

Below you will find 15 control activities that can take place during a cross-border MDRO outbreak. Can you indicate for each of these activities how likely you are to be involved in this scenario? Note: When the 'outbreak' is mentioned, this refers to the entire boundary and cross-institution outbreak, as described in the scenario.

You can read the scenario by clicking on this link.

1a Taking place in an outbreak management team / policy team to make decisions about the fight against the outbreak.

- I am definitely involved (1)
- I am probably involved (2)
- I do not know (3)
- I'm probably not involved (4)
- I am certainly not involved (5)

1b Screening ex-roommates of Mrs Schmidt who are outside the hospital (in a nursing home or at home).

- I am definitely involved (1)
- I am probably involved (2)
- I do not know (3)
- I'm probably not involved (4)
- I am certainly not involved (5)

1c Scaling up the infection prevention measures (implementing additional measures) in the nursing home.

- I am definitely involved (1)
- I am probably involved (2)
- I do not know (3)
- I'm probably not involved (4)
- I am certainly not involved (5)

1d Implementation of infection prevention measures in the home situation of the MDRO positive people (protective measures for physical care).

- I am definitely involved (1)
- I am probably involved (2)
- I do not know (3)
- I'm probably not involved (4)
- I am certainly not involved (5)

1st Information given to the MDRO positive people in the home situation.

- I am definitely involved (1)
- I am probably involved (2)
- I do not know (3)
- I'm probably not involved (4)
- I am certainly not involved (5)

1f Answering questions from the general public (not to patients involved in ring test) about the outbreak.

- I am definitely involved (1)
- I am probably involved (2)
- I do not know (3)
- I'm probably not involved (4)
- I am certainly not involved (5)

1g Exchange patient data between healthcare professionals and healthcare institutions for the purpose of outbreak control.

- I am definitely involved (1)
- I am probably involved (2)
- I do not know (3)

- I'm probably not involved (4)
- I am certainly not involved (5)

1h Communicate with the media about the outbreak
- I am definitely involved (1)
- I am probably involved (2)
- I do not know (3)
- I'm probably not involved (4)
- I am certainly not involved (5)

1i Setting up / contributing to a border and cross-institution case register of the outbreak.
- I am definitely involved (1)
- I am probably involved (2)
- I do not know (3)
- I'm probably not involved (4)
- I am certainly not involved (5)

1j Evaluating implemented actions and cooperation in cross-border and cross-institution outbreak control.
- I am definitely involved (1)
- I am probably involved (2)
- I do not know (3)
- I'm probably not involved (4)
- I am certainly not involved (5)

1k Informing municipal administrators about the outbreak.
- I am definitely involved (1)
- I am probably involved (2)
- I do not know (3)
- I'm probably not involved (4)
- I am certainly not involved (5)

1l Reporting the border and cross-institution outbreak to the national authorities (RIVM)
- I am definitely involved (1)
- I am probably involved (2)
- I do not know (3)
- I'm probably not involved (4)
- I am certainly not involved (5)

1m Contacting colleagues in Germany for exchange of information.
- I am definitely involved (1)
- I am probably involved (2)
- I do not know (3)
- I'm probably not involved (4)
- I am certainly not involved (5)

1n Contact colleagues in Germany for coordination of measures.
- I am definitely involved (1)
- I am probably involved (2)
- I do not know (3)
- I'm probably not involved (4)
- I am certainly not involved (5)

1o Contacting colleagues in Germany for coordination of communication activities.
- I am definitely involved (1)

- I am probably involved (2)
- I do not know (3)
- I'm probably not involved (4)
- I am certainly not involved (5)

1p Your opinion is important to us. Do you have comments or additions to your answers to the first part of the questions in the MDRO scenario? This can be lost here:

This was the last question in part 1. You can now start the second part of the questions on the next page.

Part 2. Cooperation between healthcare professionals
Information exchange and cooperation between healthcare institutions and healthcare professionals is important when a MDRO outbreak crosses national borders and institutions. Healthcare institutions and healthcare professionals have various roles in providing information.
The following 7 statements deal with cooperation in outbreak control.
Note: Again, when the "outbreak" is mentioned, this means the entire cross-border and cross-institution outbreak, as described in the scenario.

You can read the scenario by clicking on this link.

2a In the fight against this outbreak, I probably give advice and / or information to the following healthcare institutions / care professionals:
You can select multiple options.

2b In the fight against this outbreak, I probably get advice and / or information from the following healthcare institutions / care professionals: You can select multiple options.

2c In the fight against this outbreak, I probably work most intensively with the following healthcare institutions / healthcare professionals: You can select multiple options.

2d In the fight against this outbreak, I am probably dependent on a contribution from the following healthcare institutions / healthcare professionals: With dependent is meant that you can not continue working without the contribution of this person or organization or that the quality of your work significantly the dispute comes. By a contribution we understand for example: Information, material, advice, financial contribution, etc. You can select multiple options.

2nd Apart from outbreak control, I work together with the following healthcare institutions / healthcare professionals for other care: This means: Not specifically in the preparation for - or combating disease outbreaks, but in your daily work. You can select multiple options.

2f In my opinion, the following healthcare professional (s) should take the lead in combating the outbreak: This means that the professional (s) should take a leading role. You can also select a healthcare facility in general. The care institutions are shown in bold. You can select multiple options.

2g In my opinion, the following healthcare professional (s) should coordinate the cooperation between the various healthcare institutions and healthcare professionals during the fight against the outbreak: This means that the professional (s) should take on a facilitating role. You can also select only a healthcare institution. The care institutions are shown in bold. You can select multiple options.

2h Your opinion is important to us. Do you have comments or additions to your answers to the second part of the questions in the MDRO scenario?

This was the last question in part 2. You can now start on the third and final part of this questionnaire on the next page.

Part 3. Statements about outbreak control

Finally, there are now a number of statements about the outbreak control. Can you indicate to what extent you agree or disagree with each statement? Note: Again, when the "outbreak" is mentioned, it refers to the entire cross-border and cross-institution outbreak, as described in the scenario. You can read the scenario by clicking on this link.

3a When filling in the questions about activities in the MDRO scenario, it was clear to me in which activities I should be involved.
- Totally agree (1)
- A little bit agree (2)
- Neither agree nor disagree (3)
- A bit disagree (4)
- Disagree entirely (5)

3b It is clear to me which care institution (s) / healthcare professional (s) should take the lead in combating this outbreak.
- Totally agree (1)
- A little bit agree (2)
- Neither agree nor disagree (3)
- A bit disagree (4)
- Disagree entirely (5)

3c It is clear to me which care institution (s) / healthcare professional (s) should take a coordinating role in the fight against this outbreak.
- Totally agree (1)
- A little bit agree (2)
- Neither agree nor disagree (3)
- A bit disagree (4)
- Disagree entirely (5)

3d It is clear to me from which healthcare professionals I will receive advice or information in the fight against this outbreak.
- Totally agree (1)
- A little bit agree (2)
- Neither agree nor disagree (3)
- A bit disagree (4)
- Disagree entirely (5)

3rd It is clear to me which care professionals I will give advice or information in the fight against this outbreak.
- Totally agree (1)
- A little bit agree (2)
- Neither agree nor disagree (3)
- A bit disagree (4)
- Disagree entirely (5)

3f It is clear to me with which care professionals I will cooperate most intensively in combating this outbreak.
- Totally agree (1)
- A little bit agree (2)
- Neither agree nor disagree (3)
- A bit disagree (4)
- Disagree entirely (5)

3g I think I have sufficient capacities to act correctly in combating this outbreak.
- Totally agree (1)
- A little bit agree (2)
- Neither agree nor disagree (3)
- A bit disagree (4)
- Disagree entirely (5)

3h I think that the other healthcare professionals involved have sufficient capacities to act correctly in combating this outbreak.
- Totally agree (1)
- A little bit agree (2)
- Neither agree nor disagree (3)
- A bit disagree (4)
- Disagree entirely (5)

3i I think that the healthcare professionals involved will let go of the collective importance of the outbreak control for their own / institutional interest.
- Totally agree (1)
- A little bit agree (2)
- Neither agree nor disagree (3)
- A bit disagree (4)
- Disagree entirely (5)

3j I think that the healthcare professionals involved have the same ideas as I have of combating this outbreak correctly.
- Totally agree (1)
- A little bit agree (2)
- Neither agree nor disagree (3)
- A bit disagree (4)
- Disagree entirely (5)

3k I would like to have more clarity about the roles and responsibilities of healthcare institutions and healthcare professionals in combating border and cross-institutional MDRO outbreaks.
- Totally agree (1)
- A little bit agree (2)
- Neither agree nor disagree (3)
- A bit disagree (4)
- Disagree entirely (5)

3l Do you have comments or additions to your answers to the third part of the questions in the MDRO scenario?

This was the last question in part 3. On the next page you will find the conclusion of the questionnaire.

This is the end of the questionnaire. If you still want to check or change your answers, you can click on 'back' below. Your answers will then not be lost. Your opinion is important to us. Do you have any

other comments regarding your answers in the questionnaire, or comments about the research or the questionnaire itself? You can mention this here:

We would like to thank you for your contribution to this research. For further questions about the questionnaire or research, please contact Jacklien Maessen by e-mailing jacklien.maessen@rivm.nl Do not forget to press the blue ">>" button so that your answers are sent.
